# Normothermic Ex-vivo Kidney Perfusion in a Porcine Auto-Transplantation Model Preserves the Expression of Key Mitochondrial Proteins: An Unbiased Proteomics Analysis

**DOI:** 10.1016/j.mcpro.2021.100101

**Published:** 2021-05-23

**Authors:** Caitriona M. McEvoy, Sergi Clotet-Freixas, Tomas Tokar, Chiara Pastrello, Shelby Reid, Ihor Batruch, Adrien A.E. RaoPeters, J. Moritz Kaths, Peter Urbanellis, Sofia Farkona, Julie A.D. Van, Bradley L. Urquhart, Rohan John, Igor Jurisica, Lisa A. Robinson, Markus Selzner, Ana Konvalinka

**Affiliations:** 1 Toronto General Hospital Research Institute, University Health Network, Toronto, Ontario, Canada; 2 Division of Nephrology, Department of Medicine, Toronto General Hospital, University Health Network, University of Toronto, Toronto, Ontario, Canada; 3 Soham and Shaila Ajmera Family Transplant Centre, Toronto General Hospital, University Health Network, Toronto, Ontario, Canada; 4 Krembil Research Institute, Toronto Western Hospital, University Health Network, Toronto, Ontario, Canada; 5 Institute of Medical Science, University of Toronto, Toronto, Ontario, Canada; 6 Department of Laboratory Medicine and Pathobiology, Lunenfeld-Tanenbaum Research Institute, Mount Sinai Hospital, University of Toronto, Toronto, Ontario, Canada; 7 Department of Physiology and Pharmacology, Schulich School of Medicine & Dentistry, Western University, London, Ontario, Canada; 8 Department of General, Visceral, and Transplantation Surgery, University Hospital Essen, University Essen-Duisburg, Essen, Germany; 9 Department of Laboratory Medicine and Pathobiology, University of Toronto, Toronto, Ontario, Canada; 10 Departments of Medical Biophysics and Computer Science, University of Toronto, Toronto, Ontario, Canada; 11 Institute of Neuroimmunology, Slovak Academy of Sciences, Bratislava, Slovakia; 12 Division of Nephrology, The Hospital for Sick Children, Toronto, Ontario, Canada; 13 Program in Cell Biology, The Hospital for Sick Children Research Institute, Toronto, Ontario, Canada

**Keywords:** ischemia-reperfusion injury, kidney transplant, normothermic ex-vivo perfusion, metabolism, proteomics, systems biology, CPT2, carnitine O-palmitoyltransferase 2, mitochondrial, DCD, donation after circulatory death, DGF, delayed graft function, ESKD, end-stage kidney disease, ETFB, electron transfer flavoprotein subunit beta, FDR, false discovery rate, GO, gene ontology, NEVKP, normothermic *ex-vivo* kidney perfusion, PPAR, peroxisome proliferator-activated receptor, SCS, static cold storage, TCA, tricarboxylic acid

## Abstract

Normothermic *ex-vivo* kidney perfusion (NEVKP) results in significantly improved graft function in porcine auto-transplant models of donation after circulatory death injury compared with static cold storage (SCS); however, the molecular mechanisms underlying these beneficial effects remain unclear. We performed an unbiased proteomics analysis of 28 kidney biopsies obtained at three time points from pig kidneys subjected to 30 min of warm ischemia, followed by 8 h of NEVKP or SCS, and auto-transplantation. 70/6593 proteins quantified were differentially expressed between NEVKP and SCS groups (false discovery rate < 0.05). Proteins increased in NEVKP mediated key metabolic processes including fatty acid ß-oxidation, the tricarboxylic acid cycle, and oxidative phosphorylation. Comparison of our findings with external datasets of ischemia-reperfusion and other models of kidney injury confirmed that 47 of our proteins represent a common signature of kidney injury reversed or attenuated by NEVKP. We validated key metabolic proteins (electron transfer flavoprotein subunit beta and carnitine O-palmitoyltransferase 2, mitochondrial) by immunoblotting. Transcription factor databases identified members of the peroxisome proliferator-activated receptors (PPAR) family of transcription factors as the upstream regulators of our dataset, and we confirmed increased expression of PPARA, PPARD, and RXRA in NEVKP with reverse transcription polymerase chain reaction. The proteome-level changes observed in NEVKP mediate critical metabolic pathways. These effects may be coordinated by PPAR-family transcription factors and may represent novel therapeutic targets in ischemia-reperfusion injury.

## Highlights

• Quantitative proteomics of NEVKP and cold storage pig kidneys at three time points.
• Proteins increased in NEVKP are associated with key steps of mitochondrial metabolism.
• NEVKP attenuates proteins increased in the kidney response to injury in prior studies.
• *In silico* and mRNA analyses suggest PPAR-family members as likely regulators in NEVKP.

## Graphical abstract

**Figure:**
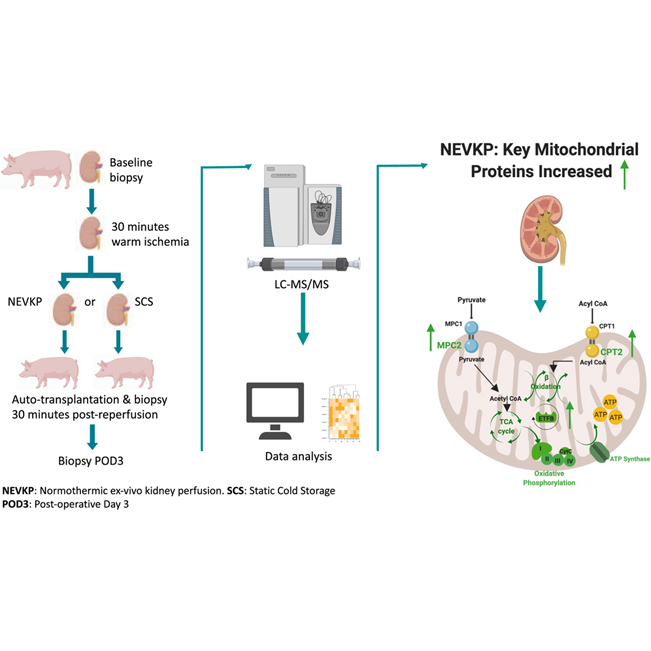

## In Brief

The molecular changes associated with normothermic *ex-vivo* kidney perfusion (NEVKP) compared with static cold storage were studied using discovery proteomics in a porcine model. NEVKP resulted in increased expression of mitochondrial proteins (ETFB, CPT2) responsible for critical metabolic steps of ATP-synthesis. PPARGC1A, PPARA/D, and RXRA were computationally predicted as upstream regulators of proteins increased in NEVKP and showed increased mRNA expression in NEVKP-treated kidneys. PPAR-family members and their target proteins may represent new therapeutic targets to ameliorate ischemia-reperfusion injury.

Kidney transplantation is considered the optimal treatment for patients with end-stage kidney disease (ESKD) (1, 2, 3, 4). The increased prevalence of ESKD in recent years has led to a growing demand for renal transplantation (5, 6), which exceeds organ supply (7, 8). Increased utilization of marginal grafts, *i.e.*, from donation after circulatory death (DCD) and extended criteria donors is incentivized in the face of organ shortage (7, 9, 10). While these organs confer a survival benefit in comparison to remaining on dialysis (10), studies have demonstrated inferior allograft outcomes compared with standard criteria donor grafts, including increased rates of primary non-function, delayed graft function (DGF), and less favorable graft outcomes at 1 year (11, 12, 13, 14, 15, 16, 17). Prolonged cold ischemic time and warm ischemic time—characteristic of DCD, are significant risk factors for these adverse outcomes. DCD kidneys, particularly, are poorly tolerant of cold ischemia and more susceptible to ischemia-reperfusion injury (IRI) (15, 16, 17, 18, 19).

The increased utilization of DCD kidneys renewed focus on optimizing organ preservation, particularly on machine perfusion alternatives to the cold anoxic storage methods (static cold storage (SCS) and hypothermic machine perfusion) currently in widespread use (20). Normothermic *ex-vivo* kidney perfusion (NEVKP) shows particular promise. While cold anoxic storage is associated with suspended cell metabolism, NEVKP provides a continuous flow of warmed, oxygenated perfusate containing nutritional substrates, thereby maintaining the metabolic activity of the tissue in a near-physiologic state (21, 22). Consequently, NEVKP permits graft assessment, conditioning, and repair throughout perfusion (23).

NEVKP results in superior short-term outcomes when compared with SCS in a porcine DCD auto-transplantation model (21, 24, 25, 26, 27, 28). Assessment of perfusion characteristics and biomarkers during NEVKP allowed prediction of post-transplant graft function (29), highlighting the potential of NEVKP to inform decision-making regarding organ suitability for transplantation.

Normothermic perfusion is successfully applied in other solid-organ transplant settings (30, 31, 32). In kidney transplantation, the first clinical trial of short (1 h) NEVKP after hypothermic preservation showed positive results (33), with further studies ongoing.

Despite the observed benefits, the molecular mechanisms responsible for improved graft function with NEVKP remain undefined. Proteins represent the functional molecules in a cell or organism, and the proteome is both highly dynamic in response to injury and modifiable by therapeutic interventions (34, 35). We and others have previously applied label-free quantification to analyze the kidney tissue proteome and defined mechanisms of injury that were not evident from gene expression changes (36, 37). Better understanding of the kidney proteome in the course of the initial injury and subsequent fast (NEVKP) or slow (SCS) recovery from IRI could lead to new insights about how kidney grafts repair themselves in the context of transplantation or potentially, any acute kidney injury. Although kidney tissue represents the main site of injury, the kidney proteome is difficult to sample longitudinally, due to the invasive nature of the biopsy and attendant risks (35, 38). Repeat kidney proteome sampling at different time points from the same animals cannot typically be applied to a small animal model and has rarely been done in a large animal model, but offers a unique opportunity to track injury over time. Similarly, metabolomic changes represent the final output of biological processes mediated by proteins, and these metabolites can both reflect protein-imposed changes and themselves modify proteins. As such, coupling the tissue proteome with metabolomic changes may uncover potentially informative indicators of the biological processes taking place in the tissue (39, 40). We hypothesized that NEVKP would induce key alterations in the renal proteome compared with SCS in a DCD model and that identifying these changes would provide insights into the molecular mechanisms associated with superior graft function in this setting. We identified the kidney tissue proteins differentially expressed between NEVKP and SCS at three time points in the evolution of warm ischemic injury and IRI. Systems analyses predicted involvement of peroxisome proliferator-activated receptors (PPAR)-transcription factors in NEVKP. Finally, we examined the potential effects of NEVKP on PPARs and PPAR-target gene expression and examined urine metabolites previously linked to PPAR activity and IRI.

## Experimental Procedures

### Experimental Design and Statistical Rationale

**Fig. 1:**
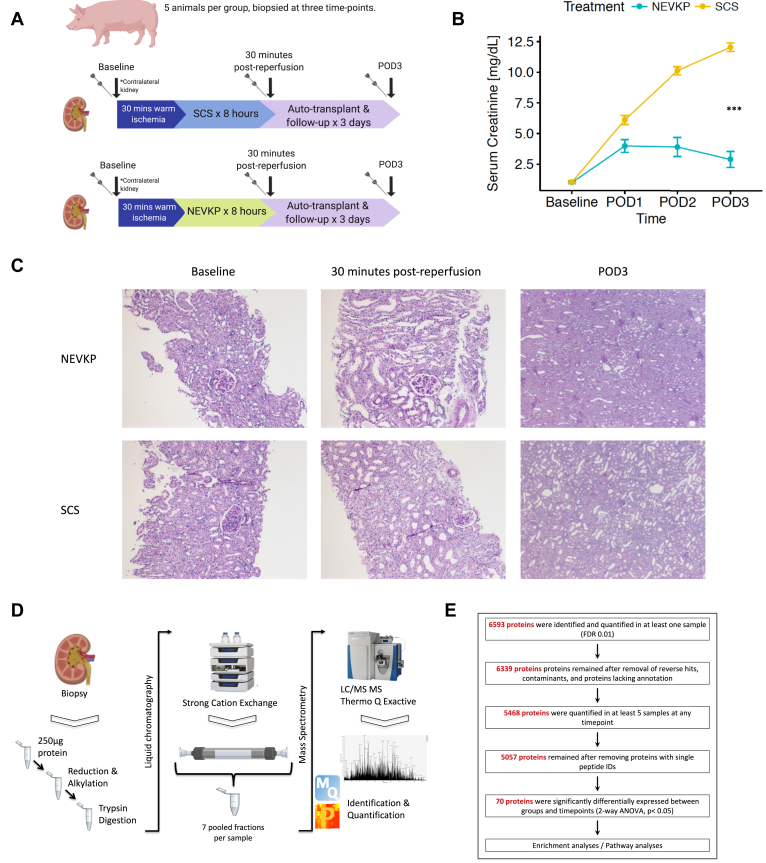
**Overview of experimental model and proteomics workflow.***A*, details of porcine DCD auto-transplantation model comprising two groups (8 h NEVKP and 8 h SCS), n = 5 animals/group; biopsied at three time points: baseline (from the contralateral kidney, prior to warm ischemia), 30 min post-reperfusion, and at sacrifice (POD3). *B*, interaction plot showing serum creatinine (mean ± SEM in mg/dl) of the transplanted animals during 3-day post-operative follow-up in NEVKP- and SCS-treated groups respectively (Data amended from reference (34)). A polynomial regression of creatinine levels in dependence on treatment, time, and time^2^ was performed (F-test, *p*-value < 2.23 × 10^−15^). *C*, light microscopy of PAS-stained images from representative NEVKP-treated (*top panel*) and SCS-treated (*bottom panel*) kidneys. Images from baseline (10×), 30 min post-reperfusion (10×), and post-operative day 3 (POD3) (2.5×) are shown. *D*, simplified proteomics workflow including sample processing, strong cation exchange liquid chromatography, and fractionation, followed by LC-MS/MS on a Thermo Q Exactive Plus mass spectrometer, and subsequent identification and quantification of peptides are shown. *E*, overview of proteomics data analysis including the numbers of identified and quantified proteins and the number of proteins differentially expressed between groups and across time points (two-way ANOVA with Tukey's HSD correction). Proteins with *q*-value < 0.05 for the effect of treatment and time were considered differentially expressed. LC-MS/MS, liquid chromatography followed by tandem mass spectrometry; NEVKP, Normothermic *ex vivo* kidney perfusion; PAS, periodic acid Schiff; POD3, postoperative day 3; SCS, static cold storage.

We conducted an unbiased proteomics analysis in a porcine DCD auto-transplantation model comprising two groups (8 h NEVKP and 8 h SCS), n = 5 animals/group. This number of animals was selected based on our prior knowledge of biological variability when performing unbiased proteomics and based on the understanding of the model from our prior work (20, 29, 41) and that of others (42, 43). Kidney biopsy tissue was collected at three time points: baseline (contralateral kidney, prior to warm ischemia), 30 min post-reperfusion, and at sacrifice (postoperative day 3 (POD3)) (Fig. 1*A*). All samples were snap-frozen in liquid nitrogen and stored at –80 °C.

There were five animals per experimental group, each biopsied at three time points (n = 30 biopsies) (Fig. 1*A*). Two biopsies with insufficient protein yield (<100 μg) to generate comparable results to the remaining biopsies were excluded. Thus, 28 biopsies (biological replicates) in total were analyzed.

### Proteomic Data Analysis

Missing values were imputed using the widely used QRILC method, which performs the imputation of left-censored missing data using random draws from a truncated distribution with parameters estimated using quantile regression with the R package imputeLCMD (v2.0) under default parametrization (44, 45). In total, 300 independent replicates of the imputed data were created. For each, we performed two-way ANOVA followed by Tukey's HSD test. The resultant *p*-values were adjusted for multiple testing by the false discovery rate (FDR) method. Finally, to obtain robust estimates of statistical significance, we calculated geometric mean of the adjusted *p*-values across the imputation replicates. Proteins whose *p*-value < 0.05 for association with the effect of treatment, time, and their interaction term were depicted by heatmap with hierarchical clustering of proteins and samples.

### Experimental Model and NEVKP

As previously described (24, 41), 3-month-old male Yorkshire pigs were used in this model. Following induction of general anesthesia, the right renal artery and vein were clamped for 30 min, mimicking a DCD-type injury. Following this, the right kidney was removed, and the vessels were cannulated and flushed with 400-mL histidine-tryptophan-ketoglutarate. The right kidney was subjected to either 8 h of SCS or 8 h of continuous pressure-controlled NEVKP, followed by auto-transplantation (24, 29). Prior to reimplantation, the contralateral kidney was removed. The pigs were followed up for 3 days following transplantation, with daily assessment of renal function, before being euthanized. The study was approved by the Animal Care Committee of the Toronto General Hospital Research Institute, Ontario, Canada. All animals received humane care in compliance with the “Principles of Laboratory Animal Care” formulated by the National Society for Medical Research.

### Sample Preparation for Proteomics Analysis

Frozen porcine kidney biopsy samples were covered with 0.1% RapiGest, followed by homogenization at 15,000 rpm for 15–30 s on the Polytron PT3100 homogenizer. Samples were subsequently sonicated for 10 s, three times, on ice. They were then centrifuged at 15,000*g* at 4 °C for 20 min. The supernatant was collected and vortexed. Total protein concentration was measured using Coomassie assay, and each sample was normalized to 250 μg of total protein. Two samples had significantly less than 100 μg of total protein and were thus eliminated from further analyses. The remaining 28 samples were analyzed in a blinded fashion. They underwent denaturation at 80 °C for 15 min, reduction with 10 mM DTT for 15 min at 65 °C, and finally, alkylation with 20 mM iodoacetamide in the dark, at room temperature, for 40 min. The samples were then incubated overnight with trypsin (Promega) 1:50 w/w at 37 °C. The following morning, trifluoroacetic acid was added to each sample at 1% v/v. Each sample was vortexed for 1 min, then left at room temperature for 5 min. The samples were subsequently centrifuged at 15,000*g* for 10 min. Supernatants were then transferred into new tubes and the pellets were discarded. Individual samples were resuspended in strong cation exchange mobile phase A (0.26 M formic acid in 5% v/v acetonitrile; pH 2–3) and loaded directly onto a 500 μl loop connected to a PolySULFOETHYL A column (2.1 mm ID × 200 mm, 5 μm, 200 Å, The Nest Group Inc.). Strong cation exchange chromatography and fractionation were performed on an high performance liquid chromatography system (Agilent 1100) using a 60-min two-step gradient. An elution buffer that contained mobile phase A with the addition of 1 M ammonium formate was introduced at 10 min and increased to 20% at 30 min and then to 100% at 45 min. Fractions were collected every 1 min from the 20 min time point onward. The resulting fractions corresponding to chromatographic peaks of eluting peptides were pooled into seven fractions, in such a way that 2–3 neighboring fractions were pooled (*e.g.*, fractions at minutes 21 + 22, 23 + 24,…, etc.).

### Tandem Mass Spectrometry

Peptides were identified by LC-MS/MS as described previously (46). Peptides from each fraction were extracted with 10 μl OMIX C18MB tips (Agilent, USA) eluted in 3 μl of 65% v/v acetonitrile, diluted to 40 μl with 0.1% v/v formic acid in pure water, and loaded onto a 3.3 cm C18 precolumn (with an inner diameter of 150 μm; New Objective), packed in-house with 5 μm Pursuit C18 (Agilent, USA). Eluted peptides from the trap column were subsequently loaded onto a resolving analytical PicoTip Emitter column, 15 cm in length (with an inner diameter of 75 μm and 8 μm tip, New Objective) and packed in-house with 3 μm Pursuit C18 (Agilent, USA). The columns were operated on the EASY-nLC system (Thermo Fisher Scientific, San Jose, CA), and this liquid chromatography setup was coupled online to Q-Exactive Plus mass spectrometer (Thermo Fisher Scientific, San Jose, CA) using a nano-ESI source (Thermo Fisher Scientific). Each fraction was run using a 60-min gradient and analyzed in data-dependent mode in which a full MS1 scan acquisition from 400 to 1500 m/z in the Orbitrap mass analyzer (resolution 70,000) was followed by MS2 scan acquisition of the top 12 parent ions. The gradient was increased from 1% to 5% Buffer B at 2 min, followed by an increase to 35% Buffer B at 49 min, 65% at 52 min, and 100% at 53 min. The following parameters were enabled: monoisotopic precursor selection, charge state screening, and dynamic exclusion (45.0 s). In addition, charge states of +1, 5–8, >8 and unassigned charge states were not subjected to MS2 fragmentation. For protein identification and data analysis, XCalibur software v3.0.63 (Thermo Fisher) was utilized to generate RAW files of each MS run.

### Protein Identification and Quantification

The raw mass spectra from each fraction were analyzed using Andromeda search engine (MaxQuant software v.1.5.3.28) against the nonredundant *Sus scrofa* database generated from a nonredundant union of 26139 porcine sequences from UniProtKB, 24556 sequences from NCBI RefSeq databases (both versions as of February 2014), and cRAP database of common contaminants (as previously published) (47). Reverse decoy mode was used. Tryptic peptides were selected with up to two miscleavages. Methionine oxidation and N-terminal protein acetylation were selected as variable modifications. Carbamidomethylation was selected as fixed modification. Protein and site FDR were set at 0.01. MS/MS parent tolerance was set to 20 ppm, and fragment tolerance was set to 0.5 Da. The minimum ratio count was set to 1. Matching between runs was selected, with a matching time window of 0.7 min and an alignment window of 20 min. Label-free quantification was performed, and normalized protein LFQ intensities were used for subsequent analyses. The data were analyzed using Perseus v.1.5.2.6. Reverse hits and contaminants were removed. Peptides and proteins with PEP >0.05 were removed. A protein was identified with >1 unique peptide. Normalized LFQ intensities were log2-transformed, and the samples were annotated according to the group (*i.e.*, NEVKP or SCS) and time point (*i.e.*, BL, 30-min post-reperfusion, POD3). We then filtered data to include only those proteins that were identified in at least five samples at any time point.

The mass spectrometry data have been deposited to the ProteomeXchange Consortium (http://proteomecentral.proteomexchange.org) *via* the PRIDE partner repository (48) with the dataset identifier PXD015277.

### Pathway and GO Analysis

Gene ontology (GO) and pathway enrichment were calculated using g:Profiler (49) and pathDIP (50), respectively. The human orthologues of the genes encoding for the 70 differentially expressed proteins were used as an input for the GO and pathway enrichment analysis. Default settings on g:Profiler (49) (https://biit.cs.ut.ee/gprofiler/gost) were used apart from the selection of Benjamini–Hochberg FDR 0.05 as the significance threshold and the exclusion of electronic GO annotations. During pathway enrichment analysis using Pathdip (50) (v3) (http://ophid.utoronto.ca/pathDIP/), we selected the extended pathway associations, integrating core pathways with experimentally proven protein–protein interactions, with the default minimum confidence level for predicted associations of 0.99 accepted.

### Measurement of Urinary Metabolites

#### Sample Preparation

Indoxyl sulfate (IS) (M-H, *m/z* 212.0018), p-cresyl sulfate (pCS) (M-H, *m/z* 187.0065), p-cresyl glucuronide (pCG) (M-H, *m/z* 283.0818), hippuric acid (HA) (M-H, *m/z* 178.0504), betaine (M + H, *m/z* 118.0868), choline (M + H, *m/z* 105.1154), carnitine (M + H, *m/z* 162.1130), and nicotinamide (M + H, *m/z* 123.0558) were quantified using ultra-performance liquid chromatography (UPLC) coupled to quadrupole time-of-flight (QToF) mass spectrometry. Urine samples were prepared by addition of ice-cold acetonitrile (3:1 acetonitrile to urine) to precipitate protein, followed by incubation at –20 °C for 20 min and centrifugation at 20,800*g* for 10 min. Acetonitrile contained chlorpropamide (4 μM) and atenolol-d7 (300 ng/ml) as internal standards. To keep analytes in the linear range of the standard curve, the supernatant from urine samples was diluted with milliQ water 40-fold for IS and pCS, 160-fold for pCG and HA, and 5-fold for betaine. The supernatant was diluted 5-fold with acetonitrile for choline.

#### Chromatography and Mass Spectrometry

IS, pCS, pCG, HA, betaine, carnitine, and nicotinamide were separated using a Waters Acquity UPLC HSS T3 column (100 mm × 2.1 mm, 1.8 μm particle size) in a Waters Acquity UPLC I-Class system (Waters). Injection volumes ranged from 0.5 to 2 μl between analytes. The mobile phase consisted of water +0.1% formic acid (A) and acetonitrile +0.1% formic acid (B) set to a flow rate of 0.45 ml/min. The UPLC gradient was as follows: 0–2 min 1%–60% B; 2–2.5 min 60% B; 2.5–3.5 min 80% B; 3.5–4.5 min 1% B. Mass spectrometry was performed using a Waters Xevo G2S-QToF mass spectrometer in negative (IS, pCS, pCG, HA) and positive (betaine, carnitine, nicotinamide) ESI modes with the following parameters: capillary voltage, 2 kV; cone voltage, 40 V; source temperature, 150 °C; desolvation temperature, 500 °C; desolvation gas flow, 1000 L/h; cone gas flow, 50 L/h. Choline was separated using a Waters Acquity BEH Amide column (100 mm × 2.1 mm, 1.7 μm particle size). The mobile phase consisted of 5 mM ammonium formate pH 3.5 (A) and acetonitrile (B) set to a flow rate of 0.45 ml/min. The UPLC gradient was as follows: 0–0.5 min 85% B; 0.5–1.5 min 85%–40% B; 1.5–2.5 min 40% B; 2.5–4.0 min 85% B. Mass spectrometry was performed in positive ESI mode with the following parameters: capillary voltage, 0.5 kV; cone voltage, 20 V; source temperature, 120 °C; desolvation temperature, 350 °C; desolvation gas flow, 1200 L/h; cone gas flow, 175 L/h. Data were acquired in sensitivity mode with a 0.05 s scan time in a 50–1200 *m/z* range and the *m/z* of each analyte was specifically targeted. Mass accuracy was maintained using a LockSpray of leucine-enkephalin (1 ng/μl) measured every 10 s with a scan time of 0.3 s and averaged over three scans.

#### Quantification

Analytes were quantified using TargetLynx V4.1 software (Waters) by comparing sample peaks to a 12-point standard curve of IS (0–1200 μM), pCS (0–300 μM), pCG (0–2000 μM), HA (0–8000 μM), betaine (0–700 μM), choline (0–700 μM), carnitine (0–300 μM), and nicotinamide (0–300 μM). Quality control samples contained known concentrations of each analyte, were prepared using the same protocol as biological samples, and injected every nine samples. The coefficient of variation of the assay was less than 10% for all analytes.

### Statistical Analysis

Significance between groups was assessed by Mann–Whitney test. *p*-values <0.05 were considered significant. Urinary metabolite concentrations were adjusted for urinary creatinine concentration. POD3 values were expressed as fold change over baseline. R (v3.5.2) and GraphPad Prism software (v8) were used for analysis and graph preparation. ∗*p* < 0.05, ∗∗*p* < 0.01, ∗∗∗*p* < 0.001.

Additional details are supplied in supplemental methods.

## Results

### Proteomic Analysis of NEVKP and SCS Biopsies

As previously reported by our group (41), NEVKP-preserved grafts demonstrated superior kidney function after heterotopic auto-transplantation compared with SCS-preserved grafts, with significantly lower serum creatinine (SCr) postoperatively in the NEVKP group compared with the SCS (Fig. 1*B*) (F-test, *p* < 2.23 × 10^−15^). Light microscopy demonstrated normal histology at baseline, with mild tubular injury in both groups at 30 min post-reperfusion, slightly more prominent in SCS (Fig. 1*C*), as previously reported in this model (41). Tubular injury and dilatation were evident at POD3 and were more severe in SCS-treated kidneys (Fig. 1*C*).

In total, 28 samples comprising nine baseline samples (four NEVKP, five SCS), nine samples from 30 min post-reperfusion (four NEVKP, five SCS), and ten samples from POD3 (five NEVKP, five SCS) were analyzed by LC-MS/MS, as summarized in Figure 1*D*.

In total, 6593 proteins were identified and quantified in ≥one sample (FDR<0.01) (Fig. 1*E*). After removal of contaminants, reverse hits, and proteins lacking annotation, 6339 proteins remained. Of these, 5468 proteins were quantified in ≥five samples at any time point. In total, 5057 proteins remained in the final dataset for analysis after removing proteins with a single peptide identification (supplemental Tables S1 and S2). Missing values were then imputed and, as expected, represented the low-abundance proteins (supplemental Fig. S1). In total, 70 proteins were identified as differentially expressed between experimental groups and time points (two-way ANOVA with Tukey's HSD post-hoc test, adjusted *p*-value < 0.05) (Table 1). These proteins were confidently identified, often with multiple peptides (Table 1).

**Table 1:** Details of the 70 proteins significantly differentially expressed between groups and across time points

Protein identifier	Pig gene	Human gene	Number of peptides identified	Time point of significance	Increased expression in	Analysis of variance (treatment∗time)
XP_005657428.1	AP1B1	AP1B1	45	30 min	SCS	0.006228671
XP_005656554.1	BOD1L1	BOD1L1	2	30 min	NEVKP	0.000195143
NP_999577.1	CYP1A1	CYP1A1	7	30 min	NEVKP	0.041408521
F1SPF6	RUVBL1	RUVBL1	11	30 min	SCS	0.04744904
XP_005674249.1	ABHD10	ABHD10	11	POD3	NEVKP	0.04502203
F1SRC5	ACO2	ACO2	53	POD3	NEVKP	0.028569884
XP_005660584.1	AIF1L	AIF1L	5	POD3	NEVKP	0.024400643
XP_003121238.3	ALDH8A1	ALDH8A1	18	POD3	NEVKP	0.036141682
F1SAM7	AMN	AMN	13	POD3	NEVKP	0.000421914
XP_005660857.1	ASRGL1	ASRGL1	12	POD3	NEVKP	0.022052418
F1SAX3	ATP1A1	ATP1A1	52	POD3	NEVKP	0.000824428
Q95339	ATP5MF	ATP5MF	3	POD3	NEVKP	0.014481044
F1SLE5	ATP6V1B1	ATP6V1B1	24	POD3	NEVKP	0.001670187
XP_003123717.3	CDHR2	CDHR2	15	POD3	NEVKP	0.018134312
XP_005659624.1	CGNL1	CGNL1	8	POD3	NEVKP	0.040245498
F1SPI0	CHCHD4	CHCHD4	5	POD3	NEVKP	0.026300633
I3LA22	CLPTM1L	CLPTM1L	4	POD3	SCS	0.020237625
I3LER5	COX4I1	COX4I1	11	POD3	NEVKP	0.012604806
NP_001233172.1	CPT2	CPT2	36	POD3	NEVKP	0.012494487
XP_005654692.1	CTTN	CTTN	9	POD3	NEVKP	0.020549915
I3LF61	CYP4F8	CYP4F8	15	POD3	NEVKP	0.014133684
XP_003125985.3	DDAH1	DDAH1	14	POD3	NEVKP	0.003389761
F1RXF3	DECR1	DECR1	16	POD3	NEVKP	0.048295598
F1SM86	EPB41L3	EPB41L3	27	POD3	NEVKP	0.017804864
XP_005665495.1	EPS15	EPS15	24	POD3	NEVKP	0.005103183
Q6UAQ8	ETFB	ETFB	17	POD3	NEVKP	0.004187732
P16549	FMO1	FMO1	26	POD3	NEVKP	0.016340051
F1S006	FN3K	FN3K	5	POD3	NEVKP	0.018266499
I3L677	G6PD	G6PD	18	POD3	SCS	5.18292E-05
F1STB6	GBA2	GBA2	19	POD3	NEVKP	0.036355338
F1S5J5	HABP2	HABP2	2	POD3	NEVKP	0.014013642
I3LTZ3	HGD	HGD	14	POD3	NEVKP	0.030812863
NP_001177098.1	HOGA1	HOGA1	12	POD3	NEVKP	0.022664251
Q06AT0	HPCAL1	HPCAL1	4	POD3	SCS	0.001994047
I3L8C5	HSPA12A	HSPA12A	31	POD3	NEVKP	0.003810733
NP_001230836.1	HSPA8	HSPA8	35	POD3	SCS	0.017815849
I3LAT6	IARS	IARS	4	POD3	NEVKP	0.000583668
F1SSR4	IVD	IVD	20	POD3	NEVKP	0.024332153
F1RU12	LACTB2	LACTB2	17	POD3	NEVKP	0.012593529
NP_001116606.1	LIPA	LIPA	8	POD3	SCS	0.003820522
I3LCC2	MARS	MARS	14	POD3	SCS	0.002446129
K7GM47	MECP2	MECP2	13	POD3	NEVKP	0.012373323
Q2EN77	MGST3	MGST3	5	POD3	NEVKP	0.031301414
F1SD56	MISP3	MISP3	11	POD3	NEVKP	0.03029979
K7GMJ2	MME	MME	43	POD3	NEVKP	0.002598281
F1SR71	MOGAT1	MOGAT1	5	POD3	NEVKP	0.009582449
XP_003355117.1	MPC2	MPC2	7	POD3	NEVKP	0.024029416
I3LMQ8	NDUFAF7	NDUFAF7	13	POD3	SCS	0.00415399
XP_005665310.1	PABPC4	PABPC4	18	POD3	SCS	0.004696561
XP_003123959.1	PDLIM4	PDLIM4	10	POD3	NEVKP	0.021981244
XP_005674442.1	PIP4K2C	PIP4K2C	8	POD3	NEVKP	0.042699072
XP_005668225.1	PLXDC2	PLXDC2	5	POD3	NEVKP	0.042880002
F2Z5L7	PSMA1	PSMA1	13	POD3	SCS	0.001007071
F1S4R1	RMDN2	RMDN2	5	POD3	NEVKP	0.02241622
F1RK77	ROGDI	ROGDI	2	POD3	NEVKP	0.024939309
F1RTJ9	RPL21	RPL21	5	POD3	SCS	0.031890423
F2Z5C7	RPS3A	RPS3A	17	POD3	SCS	0.025650357
F1RHN7	SEPT5	SEPT5	3	POD3	NEVKP	0.038733081
I3L854	SLC22A10L	SLC22A10	4	POD3	NEVKP	0.00197433
F1S5K2	SLC3A1	SLC3A1	21	POD3	NEVKP	0.007752504
B8XH67	SLC9A3R1	SLC9A3R1	26	POD3	NEVKP	0.005064438
F1SS29	SRP14	SRP14	2	POD3	SCS	5.56947E-05
XP_005662658.1	SRSF7	SRSF7	7	POD3	SCS	0.028156373
C5HGF3	TMCO1	TMCO1	2	POD3	SCS	0.005807246
XP_005672544.1	TRAPPC13	TRAPPC13	4	POD3	NEVKP	0.015800783
F1RK61	UFD1	UFD1	6	POD3	NEVKP	7.988E-05
XP_005664372.1	USP10L	USP10	9	POD3	NEVKP	0.003800781
XP_005672359.1	USP40	USP40	14	POD3	NEVKP	0.005711515
F1SK83	WASHC1	WASHC1	13	POD3	NEVKP	0.017684621
F1SRE0	XRCC6	XRCC6	12	POD3	SCS	6.28153E-05

NEVKP, normothermic ex vivo kidney perfusion; POD3, postoperative day three; SCS, static cold storage.

### Marked Differences in the Kidney Proteome at POD3

**Fig. 2:**
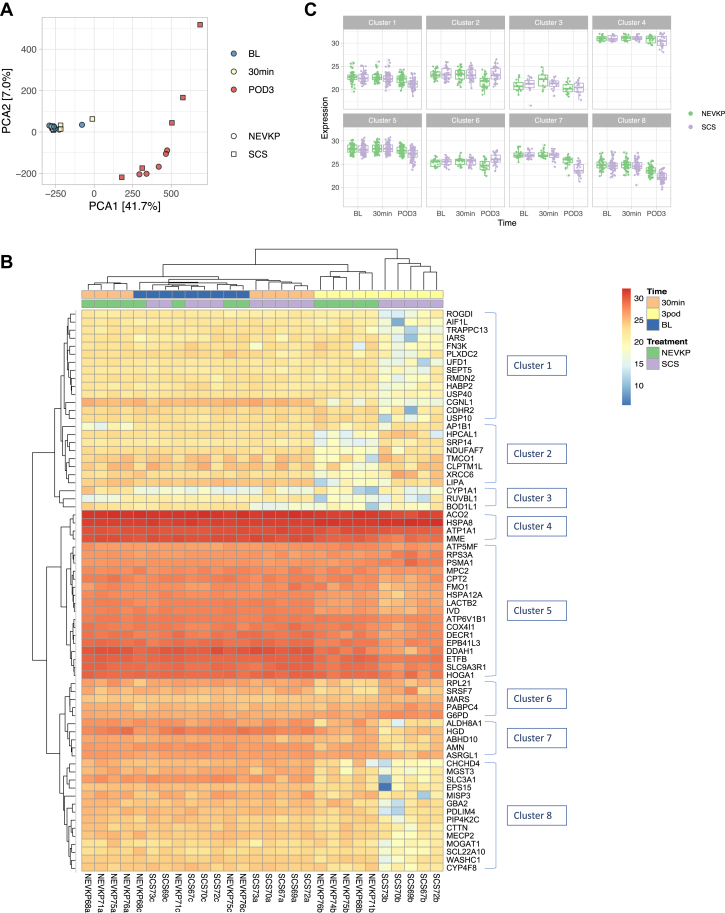
**Expression profiles of the whole dataset and of differentially expressed proteins show greatest differences between groups at POD3.***A*, principal component analysis of the proteomic dataset shows separation of POD3 samples from those at earlier time points, accounting for 41.7% of the variability in the dataset. Color denotes experimental time point and shape denotes experimental group. *B*, expression of the DE proteins across all samples depicted by heatmap with unsupervised hierarchical clustering of the proteins and samples. Columns represent each sample, and rows represent the differentially expressed proteins. The color scale indicates the LFQ abundance of the protein across all samples ranging from blue (lower abundance) to red (higher abundance). Annotation of the columns details the experimental group and time point. The dendrogram was used to identify clusters of proteins with similar expression profiles, marked on the left-hand side. *C*, Jittered boxplots showing LFQ Log2 normalized protein expression of each cluster of proteins within each group, across the experimental time points. BL, Baseline; 30 min, 30 min post-reperfusion; LFQ, normalized label-free quantification; NEVKP, normothermic *ex vivo* kidney perfusion; POD3, postoperative day 3; SCS, static cold storage.

We first examined the changes in the kidney proteome over time following IRI associated with kidney transplantation using a principal component analysis. A distinct separation was evident between POD3 samples and those taken at baseline and 30 min post-reperfusion, accounting for over 40% of the variability in the dataset. Baseline and 30 min post-reperfusion samples were intermingled, with no clear separation between groups and/or time points evident (Fig. 2*A*). Supporting this observation, the majority (66/70) of DE proteins showed significant differences in expression between the experimental groups at POD3, while 4/70 DE proteins had significantly altered expression between groups at 30 min post-reperfusion (Fig. 2*B*, Table 1). The imputed (Fig. 2*B*) and nonimputed (supplemental Fig. S2) heatmaps clustered the proteins similarly. We noted eight clusters with distinct patterns of protein expression (Fig. 2*B*). We next examined the changes in expression of the differentially expressed proteins within each group (NEVKP and SCS respectively) across the experimental time points, based on the eight protein clusters identified (Fig. 2*C*). Interestingly, the clusters enriched for metabolism-related proteins (clusters 4, 5, 7) showed that the expression of these proteins is preserved or slightly reduced in NEVKP at POD3 relative to baseline, but show a marked decrease in SCS at POD3 compared with baseline. In contrast, clusters 2 and 6 include proteins that are increased in SCS at POD3 relative to baseline, while their expression decreases in NEVKP.

### GO and Pathway Analysis

**Fig. 3:**
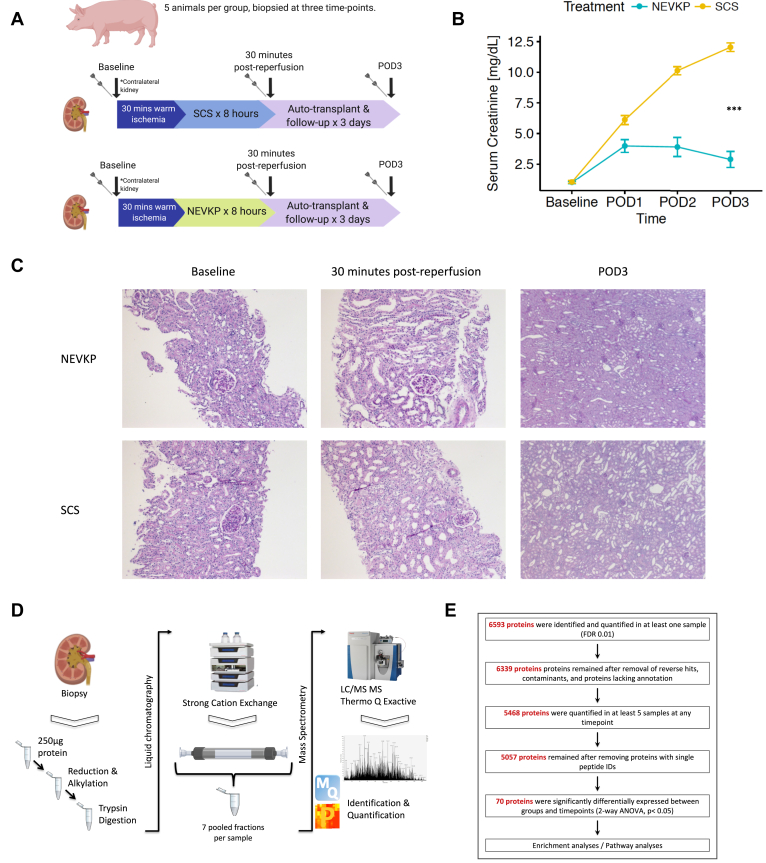
**Gene ontology and pathway analysis of dysregulated proteins.** The gene ontology terms significantly (BH-adjusted FDR < 0.05) enriched among NEVKP-increased proteins (*A*, *left*) and SCS-increased proteins (*B*, *right*) respectively. The biological pathways (literature and experimentally proved protein–protein interactions) significantly enriched (BH adjusted FDR < 0.05) among NEVKP-increased (*A*, *bottom left*) and SCS-increased (*B*, *bottom right*) proteins respectively. Node color depicts the BH-adjusted FDR as shown by the color bar; node size denotes the number of our DE proteins participating in the process/pathway in question, as shown by “# hits”; the *x* axis depicts “gene ratio,” which reflects a ratio of the number of DE proteins associated with that term: the number of DE proteins queried. BH-adjusted FDR, Benjamini–Hochberg adjusted false discovery rate; DE, differentially expressed; NEVKP, normothermic *ex vivo* kidney perfusion; SCS, static cold storage.

In total, 53/70 differentially expressed proteins were increased in NEVKP and 17 were increased in SCS (Table 1). We identified the significantly overrepresented GO terms among NEVKP-dominant and SCS-dominant proteins using g:Profiler (49). The most significant biological processes enriched in NEVKP-dominant proteins related to metabolism, specifically organic acid, amino acid, and fatty acid/lipid metabolism, and mitochondrial function (Fig. 3*A*, supplemental Table S3). Similarly, pathways significantly enriched among NEVKP-dominant proteins centered on metabolism, specifically, the tricarboxylic acid (TCA) cycle and electron transport chain (Fig. 3*A*), as determined by pathDIP (50). In contrast, SCS-increased proteins were annotated with biological processes relating to RNA catabolism and translation (Fig. 3*B*, supplemental Table S4).

Consistent with the GO analysis, pathways related to DNA replication and RNA metabolism were significantly enriched among SCS-dominant proteins (Fig. 3*B*, supplemental Table S5). Furthermore, inflammation (TNF-α and NF-kB) (51), integrin signaling (possibly mediating cell motility and extracellular matrix organization (52)), and cell cycle arrest (reported following IRI (53) and linked with inflammation and fibrogenesis (54)) were significant among SCS-dominant proteins.

### Validation of Findings Using External Datasets

**Fig. 4:**
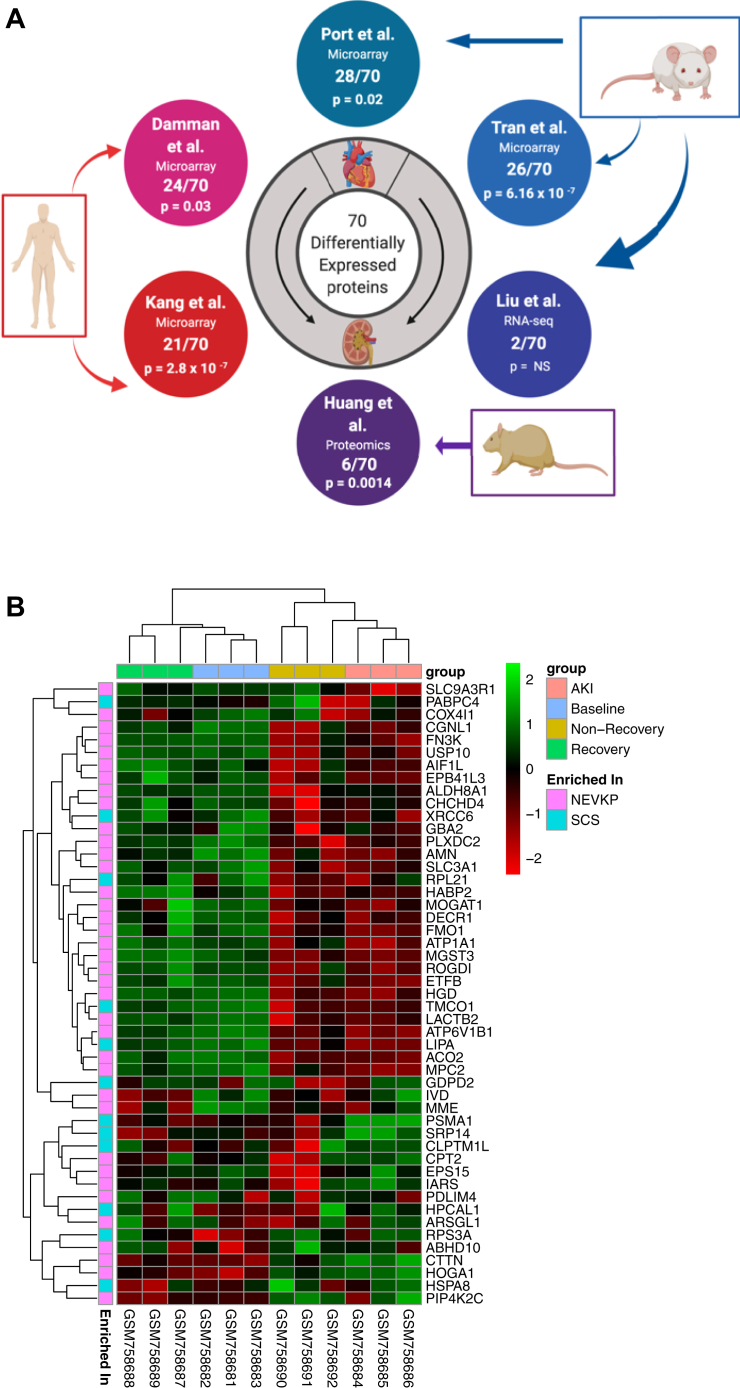
**Validation of proteomics findings in external datasets.***A*, we compared our list of DE proteins to the genes and proteins DE in a number of related studies derived from human (57, 61), mouse (55, 59, 60), and rat (56) samples, as depicted. The overlap with specific DE proteins in our study for each external study is indicated. The significance of overlap was assessed using the hypergeometric test, with resultant *p*-values shown. *B*, 49/70 of our DE proteins were represented in a mouse dataset of septic-AKI (Tran *et al.*). The heatmap depicts the expression of these 49 proteins at the gene level in the mouse dataset, using unsupervised hierarchical clustering. Columns represent the samples, and rows represent the genes, with relative expression of each gene across all samples demonstrated by pseudocolor scale ranging from –2 (red = lower expression) to +2 (green = higher expression). The columns are annotated to denote the experimental group of the mice in the Tran study. Annotation of the rows denotes increased expression in NEVKP or SCS respectively in the proteomic dataset. AKI, acute kidney injury; DE, differentially expressed; NEVKP, normothermic *ex vivo* kidney perfusion; SCS, static cold storage.

We examined our findings in relation to other relevant datasets (Fig. 4*A*, Table 2). We selected high-throughput studies relating to renal IRI as this forms the basis for the renal injury observed in our study (55, 56, 57). Importantly, Damman *et al*. (57) incorporated a cold ischemia component, analogous to SCS. As the kidneys and heart are metabolically similar (58), we included a cardiac IRI (59) study. We also included studies profiling other forms of kidney injury, specifically, septic-AKI (60), and CKD (61). We identified significant overlaps of our differentially expressed proteins with differentially expressed genes/proteins in the Port (59), Tran (60), Kang (61), Damman (57), and Huang (56) datasets respectively (Fig. 4*A*). Predominantly, expression in NEVKP opposed the perturbation observed in disease or injury. Supplemental Table S6 contains full lists of overlapping targets from each study. A subgroup of 47 differentially expressed proteins accounted for the overlap across studies (overlapping with ≥1 external study, the expression change in NEVKP opposing that observed in injury).

**Table 2:** External studies used for validation

First author	Year	Ref. No.	Organ	Organism	Specific context	Additional details	Analysis of
Liu	2017	55	Kidney	Mouse	AKI, and AKI-CKD transition	Serial profiling over 12 month period following severe bilateral IRI	Gene expression (RNA-seq)
Huang	2018	56	Kidney	Rat	AKI-IRI	Analysis of affected and contralateral kidneys at 4 and 24 h	Proteome
Damman	2015	57	Kidney	Human	Pre- and Post-Transplant	Peri-donation, post-cold ischemia, and post-reperfusion biopsies in LD, DCD and DBD donor kidneys	Gene expression (microarray)
Port	2011	59	Heart	Mouse	Myocardial Infarction	Biopsies from adjacent, non-infarcted left ventricle (or sham) at 2 days, 2 weeks and 2 months	Gene expression (microarray)
Tran	2011	60	Kidney	Mouse	AKI-Septic	Lipopolysaccharide-induced AKI. Included profiles of groups with recovery and non-recovery of renal function	Gene expression (microarray)
Kang	2015	61	Kidney	Human	CKD	Microdissected tubulointerstitial samples, control v CKD (HTN or DKD)	Gene expression (RNA-seq)

AKI, acute kidney injury; CKD, chronic kidney disease; DKD, diabetic kidney disease; HTN, hypertension; IRI, ischemia reperfusion injury.

The study by Tran *et al*. (60) permitted examination of our proteins in septic-AKI model that featured groups of mice with and without recovery of kidney function. In total, 49/70 proteins had corresponding genes in the mouse microarray. We examined the expression of these 49 genes in the mouse dataset with unsupervised hierarchical clustering of genes and samples (Fig. 4*B*). Significantly, these 49 proteins clearly separated those mice who recovered kidney function from those who did not. Mainly, the expression patterns of the proteins in NEVKP mirrored that observed in the mice at baseline and upon recovery of kidney function.

### Upstream Regulators

**Fig. 5:**
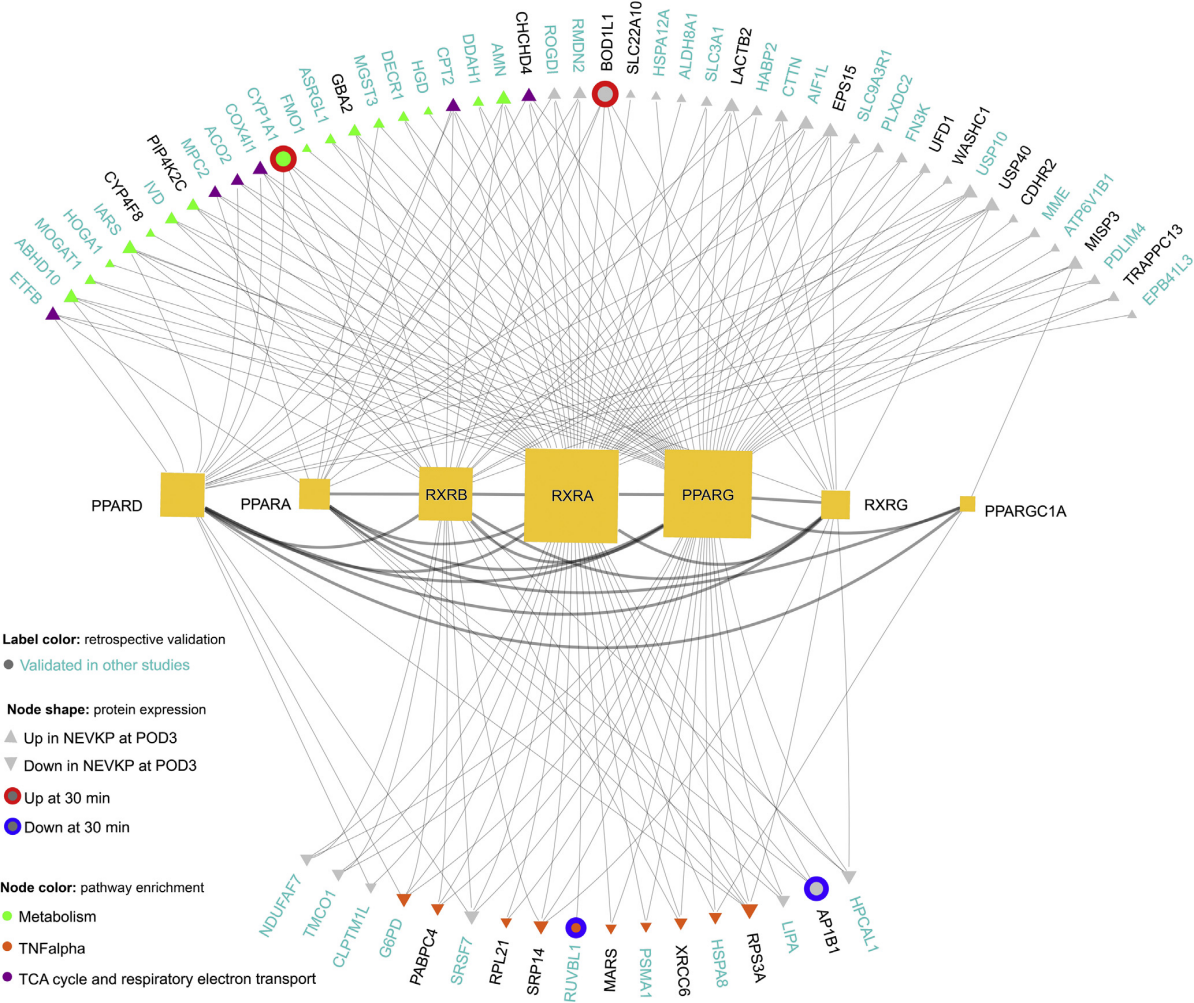
**Regulatory interactions of PPAR family members and their coactivator (PPARGC1A) and signaling partners (RXR-family members) with our DE proteins.** The regulatory interactions (*gray lines*) of PPAR family transcription factors, PPARGC1A (coactivator) and RXRs with the DE proteins in our dataset were explored using an integrated transcription factor database, CATRIN. The network image was created using the NAVIGATOR software. The size of each transcription factor node corresponds to the number of our DE proteins regulated. Among the 70 DE proteins: *red* and *blue outer circles* denote increased and decreased expression in NEVKP at 30 min post-reperfusion respectively; *gray arrowheads* reflect increased or decreased expression in NEVKP at the POD3 time point respectively. Nodes are then colored to indicate relevant pathway enrichments associated with the respective proteins. Finally, *cyan labeling* indicates those proteins that were validated in independent datasets. DE, differentially expressed; NEVKP, normothermic *ex vivo* kidney perfusion.

Our analysis suggested that preservation of key mitochondrial metabolic processes such as fatty acid oxidation (FAO) and TCA cycle/ATP-synthesis underpinned the proteome changes observed with NEVKP. The PPARs and their transcriptional coactivator PPAR-γ coactivator-1α (PPARGC1A) are viewed as the key transcription factors regulating the expression of genes involved in fatty acid metabolism and mitochondrial biogenesis. Multiple sources of evidence implicate PPARs and PPARGC1a as potential upstream regulators in our dataset. A significant overlap exists (Fig. 4*A*) between our differentially expressed proteins and the differentially expressed genes of datasets where PPARs and PPARGC1A were identified as key regulators (supplemental Tables S6 and S7) (60, 61, 62). Furthermore, using ARCHS4 (63), which integrates ChIP-seq data with large-scale RNA-seq data to predict transcription factor regulators of target genes, we verified that PPARG, PPARA, PPARD, and/or the retinoid receptor X (RXR)—the common homodimer partner for ligand-bound PPAR signaling (64, 65), were among the top-ranking transcription factors predicted to regulate 27/70 of our differentially expressed proteins (supplemental Tables S8 and S9). Finally, using CATRIN, an extended transcription factor database that integrates the findings of multiple stand-alone transcription factor databases, we demonstrated that PPAR and RXR family members were predicted to regulate 65/70 differentially expressed proteins (Fig. 5, supplemental Table S10).

### Experimental Validation of Key Findings

**Fig. 6:**
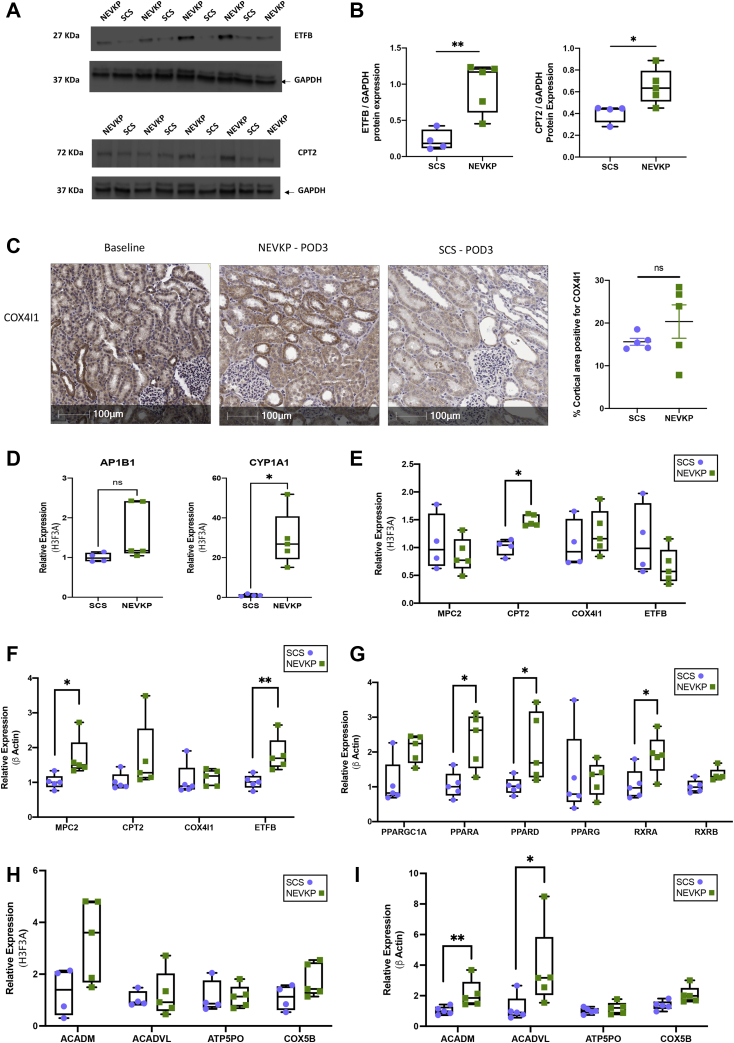
**Validation studies of differentially expressed proteins and key findings.***A* and *B*, immunoblots representing ETFB, CPT2, and GAPDH protein expression in kidney biopsy tissue from the same animals used in the proteomics analysis. Intensities for ETFB and CPT2 were measured and normalized to GAPDH using Image J software. Mann–Whitney test, n = 4–5 per group. *C*, expression of COX4I1 protein in NEVKP- and SCS-treated kidneys was verified by immunohistochemistry in new sections from POD3 formalin-fixed paraffin-embedded study samples. Magnification 20×. Scale bar 100 μm. Mann–Whitney test, n = 5 per group. *D*, relative mRNA expression of AP1B1 and CYP1A1, in pig kidneys at 30 min post-reperfusion. *E* and *F*, relative mRNA expression of genes related to the TCA cycle and differentially expressed in our dataset: COX4I1, MPC2, CPT2, and ETFB at 30 min post-reperfusion (*E*) and at POD3 (*F*) respectively. *G*, relative mRNA expression of PPARGC1A, PPARA, PPARD, PPARG, RXRA, and RXRB at POD3 in NEVKP and SCS groups. *H* and *I*, relative mRNA expression of PPAR-regulated genes ACADM, ACADVL, ATP5O, and COX5B at 30 min post-reperfusion (*H*) and POD3 (*I*) respectively in NEVKP and SCS groups. *D–I*, Mann–Whitney test, n = 4–5 per group. ∗*p* < 0.05, and ∗∗*p* < 0.01 compared with SCS. NEVKP, normothermic *ex vivo* kidney perfusion; POD3, post-operative day 3; SCS, static cold storage; TCA, tricarboxylic acid.

Given the prominence of metabolic proteins in our dataset, we selected electron transfer flavoprotein subunit beta (ETFB), carnitine O-palmitoyltransferase 2, mitochondrial (CPT2), and COX4I1 for further validation. Consistent with the proteomics findings, ETFB and CPT2 were significantly increased in POD3 NEVKP-treated kidneys in comparison to SCS-treated kidneys on immunoblotting (Figure 6, *A* and *B*, supplemental Fig. S3). Immunohistochemical analysis of COX4I1 revealed more intense staining in the tubules of NEVKP-treated kidneys, compared with SCS (Fig. 6*C*). Relative quantification of the stain confirmed this trend. We next validated our differentially expressed proteins at mRNA level. Among the proteins showing significant differences at 30 min post-reperfusion, *CYP1A1* had significantly increased gene expression in NEVKP mirroring the proteomics data (Fig. 6*D*). We examined the mRNA expression in 30 min post-reperfusion samples of a subset of mitochondrial proteins, which were differentially expressed at POD3. *CPT2* was significantly increased at this time point in NEVKP samples compared with SCS samples; however, no consistent trend was apparent for the remainder of the genes tested (Fig. 6*E*). Consistent with the proteomics data, *MPC2* and *ETFB* showed significantly increased gene expression in NEVKP at POD3, while *CPT2* and *COX4I1* expression demonstrated a similar trend (Fig. 6*F*). There were no significant differences in expression of PPAR-family transcription factors at baseline between groups (supplemental Fig. S4*A*). However, *PPARA* showed markedly increased expression in NEVKP at 30 min post-reperfusion (supplemental Fig. S4*B*). Furthermore, *PPARA*, *PPARD*, and *RXRA* showed significantly increased expression in NEVKP compared with SCS at POD3. A similar trend of increased expression in NEVKP was also evident for *PPARGC1A* and *RXRB* (Fig. 6*G*).

PPAR-family members may mediate some of their renoprotective effects by augmenting expression of the lysosomal biogenesis regulator *TFEB* (66), which was increased in NEVKP at both 30 min post-reperfusion and POD3 (supplemental Fig. S4, *C* and *D*). Finally, we examined the expression of PPAR target genes in our dataset at both 30 min post-reperfusion and POD3. A trend toward increased expression at 30 min post-reperfusion in NEVKP samples is evident for *ACADM*, *ATP5PO*, and *COX5B* (Fig. 6*H*). At POD3, both *ACADM* and *ACADVL* show significantly increased expression in NEVKP, and a similar trend is evident for *COX5B* (Fig. 6*I*).

### Urine Metabolites

IRI engenders both early and sustained alterations in the metabolic profiles of kidney tissue, plasma, and urine (56, 67, 68). We rationalized that NEVKP and SCS-induced changes identified in the proteome and transcriptome may influence the urine metabolome.

We quantified a number of metabolites in urines collected from NEVKP and SCS at each time point. Firstly, given the possible involvement of PPARs and PPARGC1A as upstream regulators of our NEVKP-proteome, we evaluated metabolites previously linked to the activity of PPARA (choline and betaine) and the renoprotective effect of PPARGC1A (betaine, choline, carnitine, and niacinamide) (62). Secondly, we were struck by the profound change in CYP1A1 at a similar, early time point following normothermic *ex vivo* perfusion in both kidney and lung (69). *CYP1A1* transcription is often viewed as a surrogate for activity of the aryl hydrocarbon receptor (70), which is linked with a number of secreted uremic toxins (including IS, pCS, pCG, and HA) that can arise in kidney injury and are measurable in urine (67, 71, 72, 73). Thirdly, we assessed lactate and glucose, which are among the metabolites increased in the urine (68), altered in tissue (67) following IRI and linked to prolonged DGF following kidney transplant (74). For the analytes successfully measured in our samples, there were no significant differences in urinary excretion at baseline between groups (supplemental Table S11). Urinary excretion of choline and betaine was increased in NEVKP compared with SCS at POD3, albeit not significantly (supplemental Fig. S5*A*). Urinary excretion of pCG and HA was significantly increased in SCS compared with NEVKP at POD3 (supplemental Fig. S5*B*). A similar (non-significant) trend was evident for IS (supplemental Fig. S5*B*).

At POD3, we observed increased urinary lactate and glucose in the SCS-treated group compared with NEVKP (supplemental Fig. S5, *C* and *D*), as observed in prolonged DGF in a cohort of DCD-transplant recipients (74).

## Discussion

This study was designed to better understand the molecular features associated with the beneficial effect of NEVKP. Our unique proteomics dataset profiles the molecular response to NEVKP and SCS following a DCD-type injury. There are three major findings: (1) proteins involved in mitochondrial energy production were significantly increased in NEVKP compared with SCS; (2) these proteins are significantly repressed in kidney disease of diverse etiologies as assessed in six external datasets; (3) PPAR and RXR transcription factors were computationally predicted upstream regulators of our metabolic proteins, and our gene expression findings support their increased activity in NEVKP.

We were struck by the observation that the differences between NEVKP- and SCS-proteomes at 30 min post-reperfusion were minor, as shown by two independent analyses. This could be explained by insufficient time to cause changes in protein translation, most changes occurring in the low-abundance proteome (typically undersampled), or that differences in response to the intervention are not driven by proteome changes at these early time points.

Our differentially expressed proteins featured critical enzymes governing mitochondrial energy metabolism. Proximal tubular epithelial cells (PTECs) utilize FAO as their preferred energy source, with inhibition of FAO associated with ATP depletion, intracellular lipid deposition, and cell death (61). PTEC lipid accumulation occurs in both AKI (62, 75, 76) and CKD (61, 77) and results in reduced oxidative phosphorylation, generation of reactive oxygen species, and kidney fibrogenesis (78). Fatty acids must conjugate with carnitine to enter the mitochondria and consequently the carnitine phosphoryltransferase enzymes (CPT1 and CPT2) represent rate-limiting enzymes of FAO (79). Of the two, CPT2 is particularly vulnerable in IRI (80). ETFB is the β-subunit of the electron transfer flavoprotein, which transfers electrons to the mitochondrial respiratory chain as FAO proceeds (81, 82). Transcriptional repression of ETFB in ischemic cardiomyopathy is described (83). Suppression of mitochondrial transcripts in proportion to the degree of kidney dysfunction is also described in other AKI models (60).

While FAO likely represents the primary means of ATP synthesis in PTECs, utilization of alternative substrates is described (84, 85), with some evidence for a glycolytic shift following IRI (86). Moreover, other metabolically active segments of the kidney have alternative substrate preferences for ATP synthesis (84, 85). Pyruvate, a hub metabolite for many metabolic pathways, enters the mitochondria *via* the mitochondrial pyruvate carrier (MPC), comprising two proteins (MPC1 and MPC2). Like PTECs, cardiomyocytes predominantly use FAO to generate ATP (87). Enhanced expression of MPC is seen in surviving myocardium post-ischemia and may mediate tissue viability in this setting (88).

The kidneys are highly metabolically active (58), requiring ATP for active solute transport against electrochemical gradients. Thus, normal kidney function is inextricably linked with mitochondrial energy production (85, 89, 90). These high energy demands may render the kidney especially vulnerable to ischemia (62, 91). We propose that preserved expression of mitochondrial metabolic enzymes in NEVKP may underpin the improved kidney outcomes observed.

CYP1A1 was increased in NEVKP at 30 min post-reperfusion, as reported after a similar *ex-vivo* perfusion period in the lungs (69). The AHR is a prominent transcriptional regulator of *CYP1A1* (70) and is potently activated by gut-derived protein-bound uremic toxins, which accumulate in plasma and tissues in AKI and CKD (72, 73, 92, 93). This activation is linked with the vascular dysfunction and systemic inflammation of CKD (72, 94, 95, 96). In our study, these toxins were increased in urine of SCS pigs, potentially linking to the inflammatory pathways of SCS. AHR-independent pathways also regulate CYP1A1 expression (70, 97, 98, 99) including PPARA (100). CYP1A1 has well-described roles in drug metabolism and lipid oxidation (98). CYP1-enzymes participate in the oxidative biosynthesis of polyunsaturated fatty acids (101), and the specialized proresolving lipid mediators (SPMs) derived from these precursors (102). SPMs actively coordinate the resolution of acute inflammation, thereby limiting the inflammatory response (103, 104). Analysis of peritonitis-associated lipid-mediator metabolomes in CYP1-family knockout mice revealed increased neutrophil recruitment, elevated leukotrieneB4, and reduced intermediary compounds of SPM biosynthesis (105). The induction of CYP1A1 in NEVKP may reflect these non-classical, pro-resolving pathways of activation.

PPAR-family members and their transcriptional coactivator PPARGC1A emerged as likely upstream regulators in our dataset, with *PPARA* showing increased expression at 30 min post-reperfusion in NEVKP, and *PPARA/D* and *RXRA* showing significantly increased expression in NEVKP at POD3. The renoprotective effects of PPARs and PPARGC1A, particularly, have been described in models of septic (60, 106), toxic (66, 107), and ischemic (62, 108, 109) AKI. Downregulation of PPARGC1A and related transcripts is observed in CKD of diverse etiologies (61, 110) and implicated in the development of inflammation (111) and age-related fibrosis in the kidney (112). Kidney transplants with increased PPARGC1A expression demonstrated a faster and more complete recovery from DGF (113). PPARGC1A is considered the “master regulator” of mitochondrial biogenesis, binding to a host of transcription factors (most notably PPAR-family members) to increase expression of genes that augment mitochondrial abundance, oxidative phosphorylation, and FAO (114, 115, 116, 117). Observations that tubular PPARGC1A can reduce the severity of AKI and accelerate functional resolution (62, 66, 108, 118) are consistent with the high metabolic activity of PTECs (119). Less metabolically active kidney cell types including endothelial cells (62) and podocytes (120, 121) may not experience the same benefit, suggesting a cell-type-specific role for PPARGC1A in the kidney.

Previous observations about the metabolic footprint of PPARGC1A renoprotection (62) prompted us to examine related markers in the urine. A modest increase in urinary choline was evident in the NEVKP-treated group. Choline and betaine are renal osmolytes (122). Increased urinary osmolytes are reported following cold ischemia and hypothesized to reflect medullary cell damage (123). Increased urinary betaine and choline are reported in CKD (124) and incipient diabetes (125). Conversely, other evidence suggests that our observed increases in urinary choline could reflect increased PPAR activity (62). Increased concentrations of choline are noted in the kidneys of wild-type mice in comparison to PPARA^−/−^ mice (126). Treatment of healthy individuals with fibrates (PPARA-agonists) results in increased urinary choline and betaine (127), with similar findings in animal models (128). Our urinary observations support our proteomic and gene level findings, which together suggest that the alterations observed in NEVKP-treated kidneys may reflect increased PPARA and PPARGC1A activity.

Similarly, decreased lactate excretion may be indicative of diminished lactate production and diminished glucose utilization in glycolysis at the tissue level in NEVKP compared with SCS and increased oxidative phosphorylation in NEVKP. This would be consistent with our observations of increased mitochondrial enzymes involved in oxidative phosphorylation in NEVKP.

Our study has many strengths. Given the anatomical and physiological similarities of pigs and humans, our large animal model is readily clinically translatable and well suited to the study of IRI and transplantation. In contrast to previous studies (69, 129), we assess the impact of NEVKP posttransplant and examine the functional significance of *ex-vivo* observations. Our systems biology approach incorporates transcriptomic and targeted metabolomic analyses, as well as an analysis of upstream regulators. Finally, this is a novel dataset; to our knowledge, this is the first proteomics study related to NEVKP. Notwithstanding the strengths of our study, some limitations exist. Our porcine DCD model lacks some elements typically observed clinically, most notably severe antecedent illness in the donor, alloantigen exposure, and postoperative immunosuppression. The structural and functional annotation of the pig genome remains incomplete (130), rendering biological interpretation challenging. While our differentially expressed proteins were predicted to be regulated by PPAR/RXR transcription factors, which was supported by their alteration at mRNA level, it is plausible that post-translational modifications contributed to differences in protein abundance. Lastly, while the central conclusion of our analysis describes preserved mitochondrial function related to NEVKP, direct visualization of mitochondria on a cellular level is lacking, and further studies will seek to assess mitochondrial structure and function directly in this model. Likewise, future work will attempt to uncover the relative contribution of normothermia and oxygenation respectively to the beneficial effects of NEVKP. Future studies will be also designed to examine the cause-and-effect relationship between these proteins and transcription factors and the renal outcomes post-NEVKP.

**Fig. 7:**
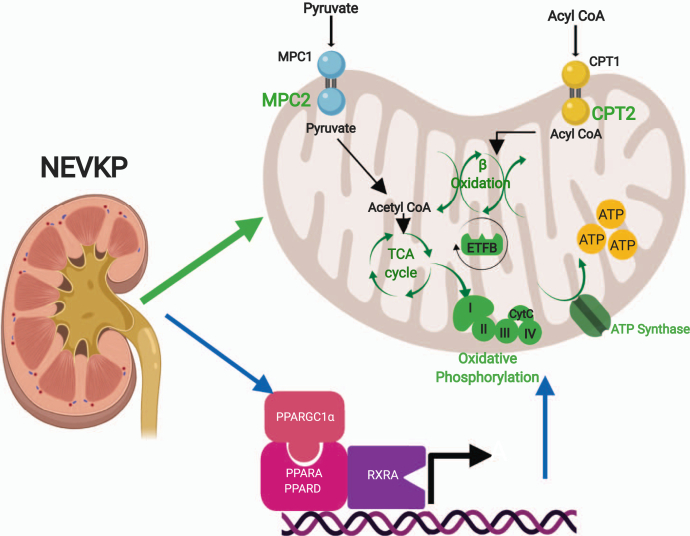
**Proposed role of NEVKP in attenuating ischemia-reperfusion injury in a DCD-model of auto-transplantation.** NEVKP is associated with preserved expression of proteins mediating critical metabolic processes in the mitochondria in comparison to SCS. We demonstrate increased expression of proteins mediating the entry of key energy-producing substrates into the mitochondria (MPC2, CPT2), proteins involved in the TCA cycle (ACO2), electron transfer (ETFB), oxidative phosphorylation (COX4I1), and ATP synthesis (ATP5MF) resulting in enrichment of fatty acid β-oxidation, the TCA cycle, and oxidative phosphorylation. NEVKP results in increased urinary choline posttransplant and decreased urinary glucose and lactate in comparison to SCS. All NEVKP-increased processes are shown in *green*. The *blue arrows* represent our findings on gene expression that these effects are centrally regulated by members of the PPAR-family of transcription factors (*PPARA* and *PPARD*), *RXRA*, and their transcriptional coactivator *PPARGC1a*. NEVKP, normothermic *ex vivo* kidney perfusion; SCS, static cold storage; TCA, tricarboxylic acid.

In summary, we present a detailed analysis of the changes in the kidney proteome induced by NEVKP in comparison to SCS. We conclude that preservation of key mitochondrial enzymes mediating crucial metabolic pathways may be responsible for the superior kidney outcomes seen with NEVKP and that these effects may be, in part, coordinated by PPAR/RXR transcription factors (notably PPARA/D and RXRA) and the coactivator PPARGC1A (Fig. 7). Our findings suggest potential therapeutic targets to ameliorate IRI in kidney transplantation.

## Data availability

The data supporting the findings of this study have been deposited to the ProteomeXchange Consortium (http://proteomecentral.proteomexchange.org) with the dataset identifier PXD015277.

## Supplemental data

This article contains supplemental data (24, 27, 28, 41, 55, 56, 57, 59, 60, 61, 63, 131, 132, 133, 134).

## Conflict of interest

The authors declare no competing interests.
