# Supplementary material for: Normothermic Ex-vivo Kidney Perfusion in a Porcine Auto-Transplantation Model Preserves the Expression of Key Mitochondrial Proteins: An Unbiased Proteomics Analysis
Source: Mol Cell Proteomics. 2021 May 23;20:100101. doi: 10.1016/j.mcpro.2021.100101 (PMC8253910; doi:10.1016/j.mcpro.2021.100101)
Supplement: Supplemental material [file mmc14.pdf]

Supplemental Material Table of Contents

Supplemental Methods

Supplemental Figure Legends

Supplemental Figures

SF1: Distribution of protein abundance with QRILC-imputed values

SF2: Heatmap depicting the expression profiles of the DE proteins using non-imputed data

SF3: Uncropped western blot images

SF4: Gene expression of PPAR-family and related transcription factors

SF5: Analysis of Urine Metabolites

Supplemental Tables

ST1: Peptide File

ST2: Protein Annotation and Expression

ST3: GO analysis of NEVKP-dominant proteins

ST4: GO analysis of SCS-dominant proteins

ST5: Pathway analysis (pathDIP)

ST6: Summary table of the validation with external datasets

ST7: Overlap of DE proteins with PPARGC1A-regulated IRI dataset

ST8: ARCHS4 analysis- Ranking of top transcription factors

ST9: ARCHS4 analysis- z scores of PPAR-family transcription factors in genes encoding DE Proteins

ST10: Network mapping using CATRIN relating to Figure 5.

ST11: Urinary metabolite concentrations adjusted by urinary creatinine

ST12: Primer sequences used in RT-PCR

ST13: Details of NEVKP perfusate

Supplemental Methods

Experimental model

As previously described,[1,2] 3 month old male Yorkshire pigs were used in this model. Following induction of general anesthesia, the right renal artery and vein were clamped for 30 minutes, mimicking a DCD-type injury. Following this, the right kidney was removed, and the vessels were cannulated and flushed with 400-mL histidine-trytophan-ketoglutarate. The right kidney was subjected to either 8 hours of SCS or 8 hours of continuous pressure-controlled NEVKP, followed by auto-transplantation. Prior to re-implantation, the contralateral kidney was removed. The pigs were followed up for 3 days following transplantation, with daily assessment of renal function, before being euthanized. The study was approved by the Animal Care Committee of the Toronto General Research Institute, Ontario, Canada. All animals received humane care in compliance with the "Principles of Laboratory Animal Care" formulated by the National Society for Medical Research.

NEVKP

Normothermic ex vivo perfusion was conducted as previously described.[1-3] The perfusion circuit is based on modified neonatal cardiopulmonary bypass technology. A centrifugal pump propels the perfusion solution into the oxygenator, where it is oxygenated and warmed to 37 °C. After passing the arterial filter (95% O_2, 5% CO_2, 2L/min), the perfusate is driven with a pressure of 65 mm Hg through the renal artery into the graft which is housed in a chamber. The venous outflow leads the perfusate back into the venous reservoir. Any urine produced is collected throughout the perfusion. As previously described, the perfusion circuit was primed with Ringer's lactate (200mL), Steen solution TM (150mL, XVIVO Perfusion AB, Goteborg, Sweden), washed leukocyte-filtered erythrocytes (125mL), double reverse osmosis water, 8mL sodium bicarbonate (8.4%), 1.8mL calcium gluconate (10%, 100 mg/mL), and heparin to achieve a near physiologic perfusate composition.[4] During perfusion, urine output was replaced with Ringer's lactate. Verapamil (0.25 mg/h) was administered intra-arterially, and amino acids and glucose (1 mL/h) with insulin (5 IU/H)

were administered continuously during the perfusion and were adapted to maintain perfusate glucose level between 5 and 15 mmol/L, (**Supplemental Table 13**). Blood gas parameters were measured hourly, with optional administration of bicarbonate to maintain acid-base homeostasis, though this was typically not required after the first hour of perfusion.[3]

Sample collection and storage

18G core biopsies were taken at the first two timepoints. At POD3 a wedge biopsy was reserved. Urine samples obtained from bladder puncture were immediately centrifuged (9,000*g* x 10 mins) and the supernatant reserved. All samples were snap-frozen in liquid nitrogen, then stored at -80°C.

Upstream regulator analysis and network visualization

The ARCHS4 pipeline has aligned and curated the majority of published RNA-seq data (GEO/SRA) from human and mouse. The ARCHS4 web interface makes this information available at the gene and transcript levels. Additionally, the web resource enables exploration of the processed data through a number of querying tools, including the prediction of upstream transcription factors based on prior knowledge and co-expression with identified targets as determined by ChIP-seq data from the ChEA and ENCODE gene set libraries. We interrogated ARCHS4[5] to identify transcription factors predicted with a high likelihood (i.e. the top 10 ranking factors with z-score >2) to regulate genes encoding our differentially expressed (DE) proteins. The resultant list of transcription factors was then ranked on the number of our DE proteins they were predicted to regulate (shown in **Supplemental Table 8 and 9**)

Catrin (http://142.150.188.233:9080/Catrin/index.jsp), is a **Ca**talogue of **T**ranscriptional **R**egulatory **In**teractions that integrates 15 separate transcription factor databases. Many of these databases utilize different methods to identify transcription factor to gene pairs, and some of the databases focus only on a single transcription factor or on a single class of transcription factors. For this reason, the overlap across databases is quite poor, and the integration of such data extends the coverage of

transcriptional regulatory interactions. DE proteins were used to query Catrin with all resources except TF2DNA.experimental. Regulatory interactions were obtained from specified transcription factors (PPARA, PPARD, PPARG, PPARGC1A, RXRA, RXRB, RXRG) to query genes. Due to the completeness of Catrin data, interactions retrieved from it were used to build a network. Network visualization analysis was performed using NAViGaTOR 3.0.10, a stand-alone scalable software designed to create, annotate, visualize and analyze networks.[6]

The selection of PPAR- and RXR- family regulated genes for Figure 6 was also made using CATRIN.

Analysis of external datasets

We explored the context of our findings in relevant external datasets by examining for overlap between our 70 DE proteins and the DE proteins or transcripts in the external studies (**Supplemental Table 6**).

Damman et al.[7] examined gene expression in deceased donor biopsies, at three timepoints: retrieval, after cold ischemia, and 45-60 minutes post-reperfusion. Living donor kidney biopsies at two timepoints: before clamping of the renal artery, and 45-60 mins post-reperfusion were also examined. Using the detailed annotation supplied with this study on GEO2R (GSE43974) we identified the genes DE (FDR-adjusted $p < 0.05$) at the post-reperfusion timepoint between DCD donors who experienced DGF, and living donors who did not (viewing this as the most analogous comparison to SCS- and NEVKP-kidneys respectively).

Tran et al.[8] studied the gene expression profiles of mice subjected to a septic-AKI induced by lipopolysaccharide. In this experiment, all mice received fluid resuscitation after developing the AKI; some then recovered baseline renal function, while others did not. Using the annotation details provided (GSE30576 - GPL8759) on GEO, we identified the genes DE (FDR adjusted $p < 0.05$) between mice with an AKI in comparison to baseline. Separately, we downloaded the raw data files for this experiment (GSE30576 -GPL8759), and after pre-processing and Log_2 normalization (Limma

v3.34.14)[9], generated an expression dataset for all animals (Baseline, AKI, Recovered, and Non-recovered). We extracted the values for the genes corresponding to the 70 DE proteins from our proteomics dataset. In total, 49 of the proteins were represented in the murine dataset. The expression profiles of these genes across all samples was plotted using a heatmap (pheatmap v1.0.12) with unsupervised hierarchical clustering of genes and samples.

Kang et al.[10] performed RNA sequencing in micro-dissected tubulointerstitial samples to identify genes DE between individuals with CKD and healthy controls. Details of DE transcripts are supplied in their supplement.

Liu et al.[11] used RNA-seq at multiple timepoints to report the temporal-specific alterations in gene expression following bilateral severe IRI, and the ensuing progression to CKD. The list of genes DE in these mice when compared to controls, is supplied in their supplement. Huang et al[12] performed a LC-MS/MS proteomic analysis of rat kidney cortices at 4 and 24 hours post-reperfusion following a 45-minute period of unilateral IRI, using contralateral kidneys and healthy controls as comparators. The list of DE proteins is provided in their supplement.

Port et al[13] examined gene expression in adjacent but non-infarcted left ventricle of mice in a model of myocardial infarction at three timepoints (2 hours, 2 days and 2 weeks post-infarction), using sham-operated time-matched controls as controls. The list of DE genes identified in this study is provided in their supplement.

For each study, the statistical significance of the overlap identified was assessed using the hypergeometric test in R.

Gene expression

Total RNA was extracted from frozen wedge and core biopsies of pig kidney tissue using the RNAeasy Mini Kit (Qiagen). RNA concentration was measured in each sample using Nanodrop (Thermo Scientific), and 500ng of RNA were subsequently retrotranscribed to cDNA using the High Capacity cDNA Reverse Transcription Kit (Applied Biosystems). Gene expression of ACADM, ACADVL,

AP1B1, ATP5PO, COX4I1, COX5B, CPT2, CYP1A1, ETFB, MPC2, PABPC4, PPARA, PPARD, PPARG, PPARGC1A, RXRA, RXRB, and TFEB was determined by real-time quantitative PCR using Power SYBR® Green PCR Master Mix (Applied Biosystems) in a StepOne Plus System (Applied Biosystems). For each time point, gene expression data were normalized to the most stable housekeeping gene across conditions: RPS16 (baseline), H3F3A (30 min), and ACTB (POD3). Primer sequences are summarized in **Supplemental Table 12**.

Immunoblotting

Fresh protein lysates were made from stored residual biopsy tissue. After protein quantification (Micro BCA Protein Assay kit (ThermoFisher Scientific)), 10µg of protein per well was loaded onto 10% acrylamide gels (BioRad) and separated by SDS-PAGE. Membranes were incubated with antibodies to CPT2 (26555-1-AP, Proteintech®), ETFB (LS-C81860 / 146214. GAPDH (CB-1001, Millipore Sigma) was used as a loading control. Secondary antibodies included: HRP-conjugated anti-mouse (P044701-2, Agilent) and HRP-conjugated anti-rabbit (A0545, Sigma). Western blot images were acquired using the DNR Bioimaging Systems MicroChemi 4.2. which incorporates the molecular weight reference image with the image of the membrane.

Following detection, bands were quantified by densitometry using Fiji/Image J (1.x).[14,15] For validation, 4-5 animals per group were studied.

Immunostaining

For COX4I1 immunohistochemistry, antigen retrieval was performed in dewaxed and rehydrated 5µm sections by heating the samples with a citrate buffer (pH6) (Abcam) in a pressure cooker for 20 minutes. Sections were then incubated with rabbit anti-COX4I1 antibody (dilution 1:100, MA-15078, Invitrogen) diluted in Dako diluent (Dako, No.S0809). HRP- conjugated anti-rabbit (MP-7401, Vector Labs) was used as secondary antibody. Samples were further incubated with DAB, then

counterstained with hematoxylin to visualize nuclei. Slides were digitally scanned in a ZEISS Axio Scan.Z1 system.

Cortical areas on the whole slide image (wedge biopsy, POD3) were assessed for positive staining. Areas of capsule, medulla, artefact and large blood vessels were excluded from analysis. To assess protein expression, the percentage of positive staining in the cortical area assessed was quantified using the Halo software (Indica Labs, version 3.0.311).

Urine Biochemistry and Creatinine measurements:

The BioProfile FLEX analyzer (novaBiomedical) was used to measure glucose and lactate from a 300μl of urine, as per the manufacturer's instructions. Urine creatinine was quantified using the colorimetric Creatinine Assay Kit (ab204537, Abcam plc) as per the manufacturer's instructions.

Supplemental Figure Legends

Supplemental Figure 1: Histogram depicting the distribution of protein abundance with the QRILC imputed values. The imputed values are shown in light grey, while the measured ones are shown in dark grey. As expected, the imputed values represent low abundance proteins.

Supplemental Figure 2: Heatmap generated using unsupervised hierarchical clustering depicting the expression profiles of the 70 DE proteins using the non-imputed data (with missing values assumed to be "0"). Row annotations highlight distinct clusters of proteins exhibiting co-expression across samples. Annotation of the columns details the experimental group and timepoint.

Supplemental Figure 3: Full western blot images. Western blot images were acquired using the DNR Bioimaging Systems MicroChemi 4.2. which incorporates the molecular weight reference image with the image of the membrane.

Supplemental Figure 4: Relative mRNA expression of PPAR-family transcription factors at baseline (A) and 30 minutes post-reperfusion (B). Relative mRNA expression of TFEB at baseline and 30 minutes post-reperfusion (C, D) Significance assessed by Mann Whitney test, n=4-5 per group.

Supplemental Figure 5: Analysis of Urine Metabolites

The absolute urinary concentration of **(A)** Betaine and Choline and **(B)** Indoxyl Sulfate, Hippuric Acid, p-Cresyl Glucuronide, p-Cresyl Sulfate were measured (μmol/L) and normalized to urinary creatinine (μmol/L). Values at POD3 are expressed as fold change over the baseline value. N= 5 urines per group. Urinary concentration of **(C)** Glucose and **(D)** Lactate were measured (both mmol/L) at POD3, and normalised to urinary creatinine (μmol/L). N=3-4 urines per group. Differences between groups were assessed by Mann-Whitney test, *p<0.05 compared to SCS at POD3. NEVKP, normothermic ex vivo kidney perfusion; POD3, post-operative day 3; SCS, static cold storage.

Supplemental Figures 1-5

Supplemental Figure 1

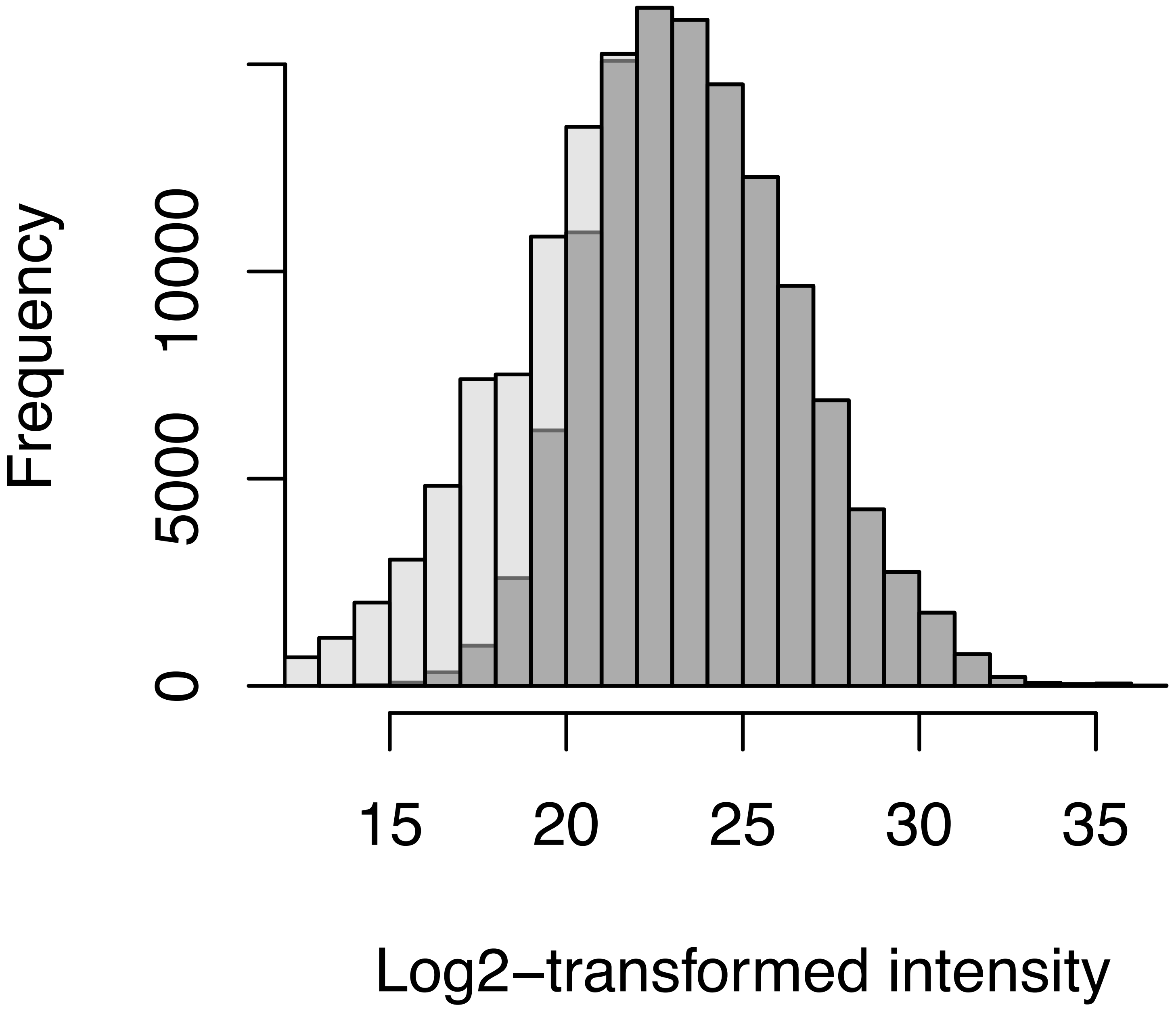

Supplemental Figure 2

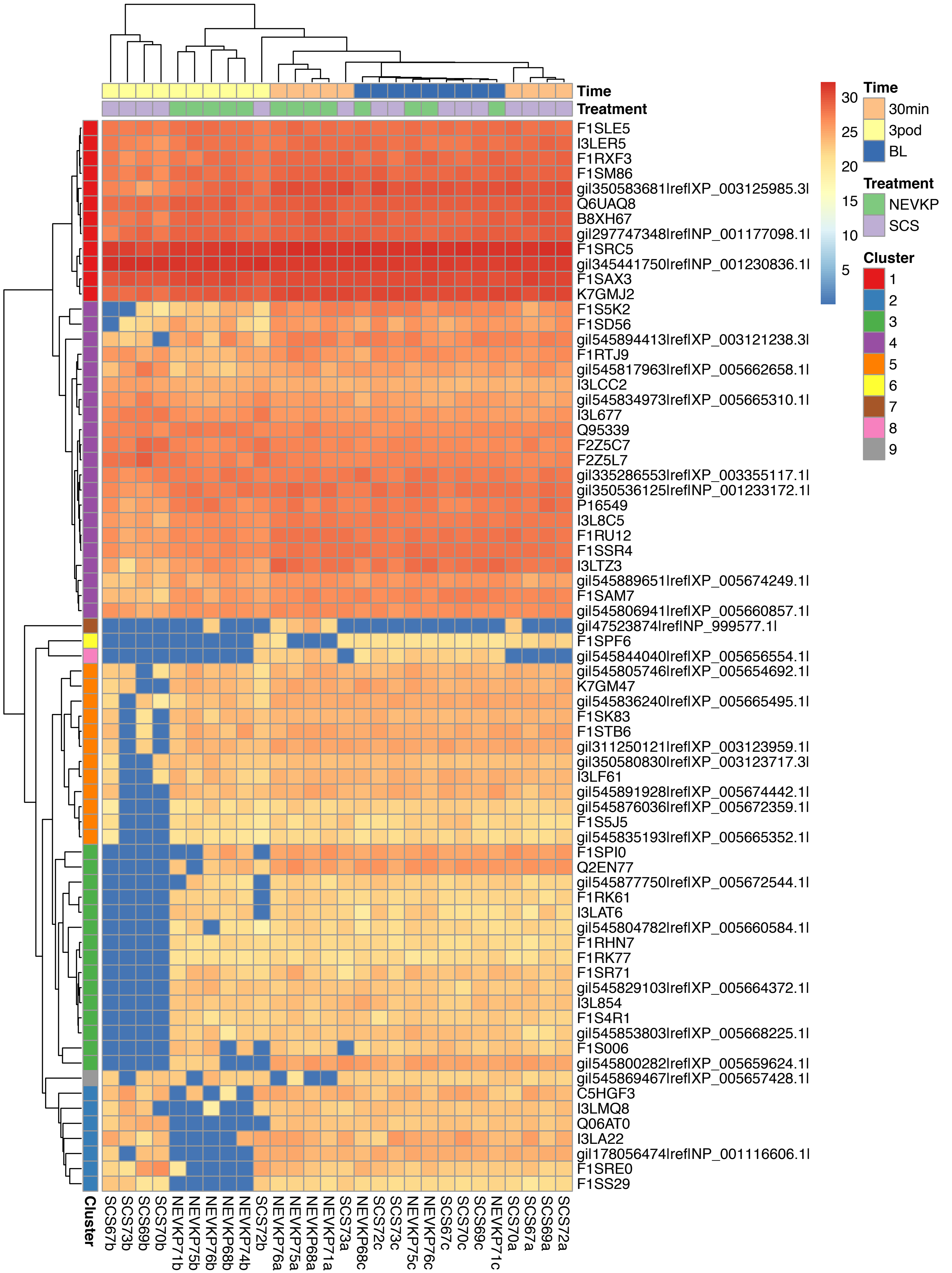

Supplemental Figure 3

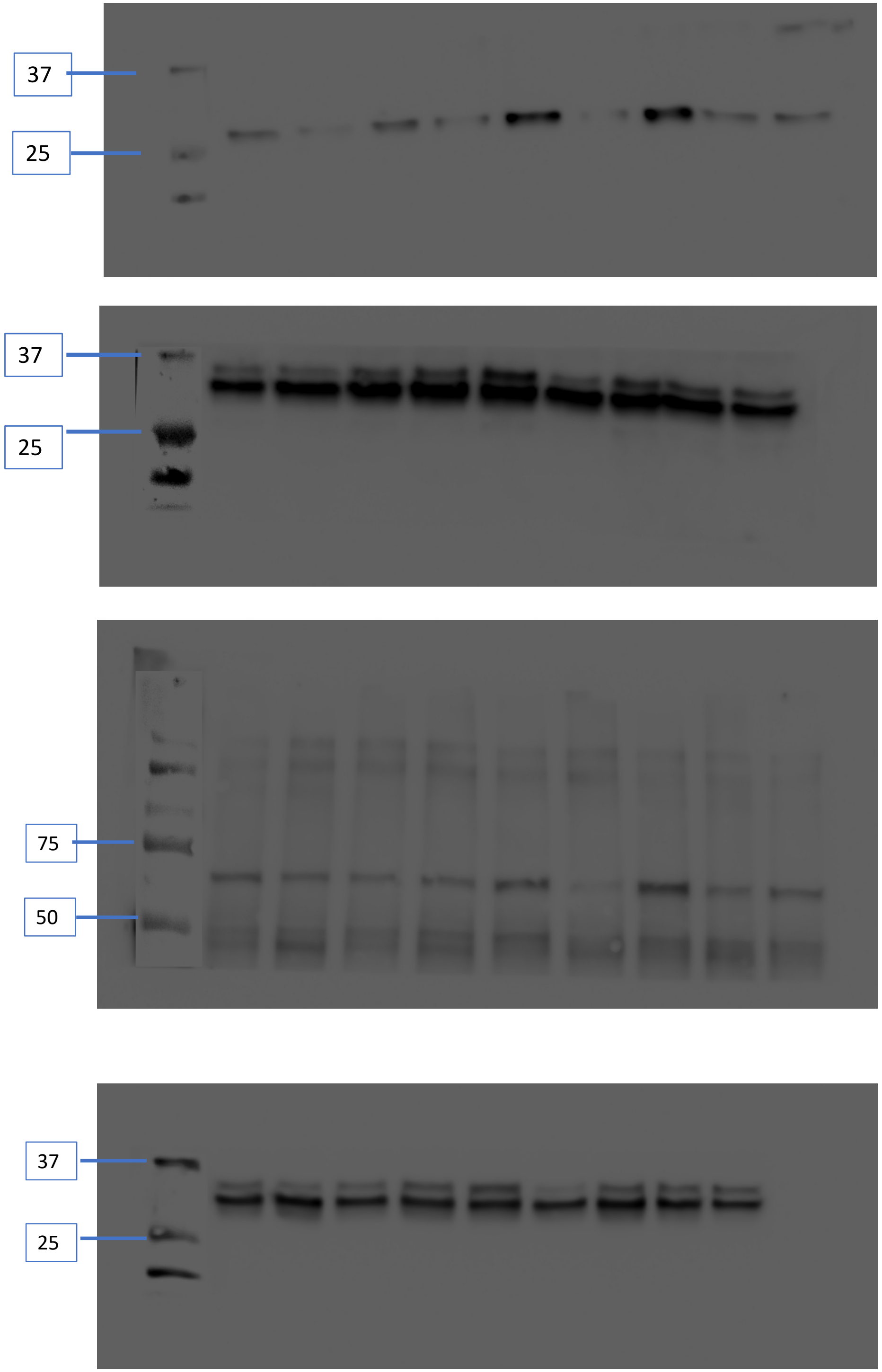

Supplemental Figure 4

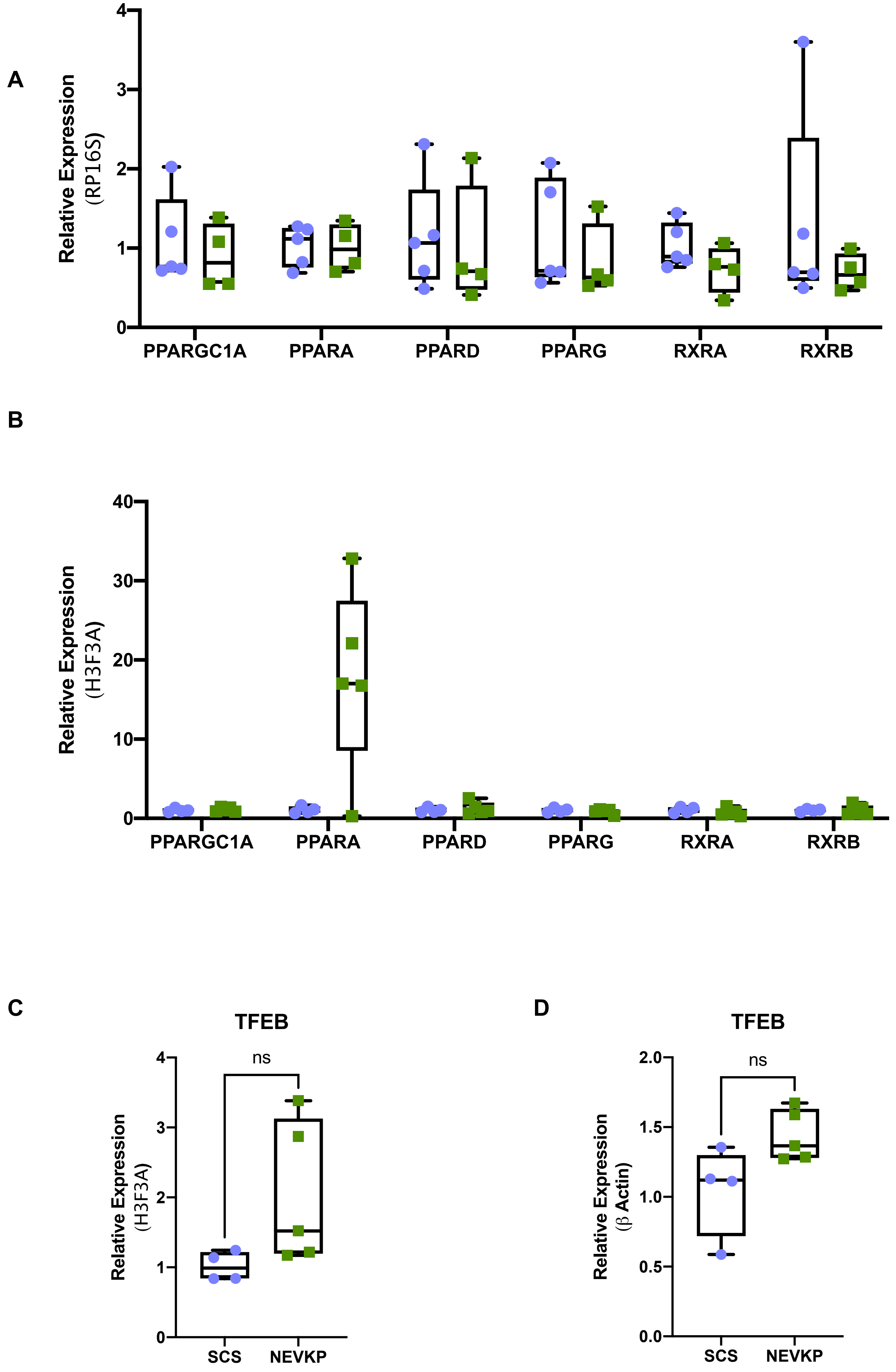

Supplemental Figure 5

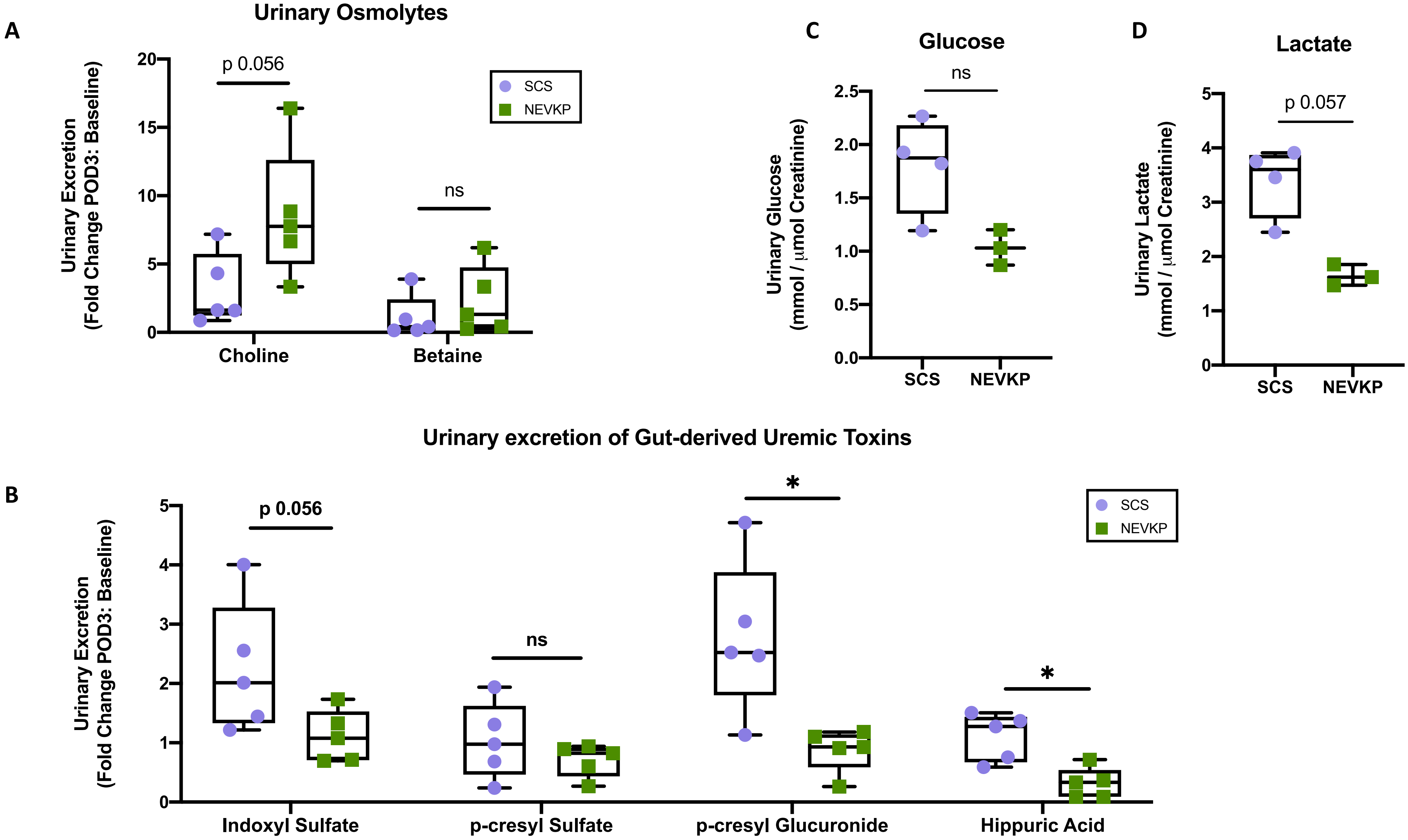

References

1. Kaths, J.M., *et al.* Normothermic Ex Vivo Kidney Perfusion for the Preservation of Kidney Grafts prior to Transplantation. *J Vis Exp*, e52909 (2015).
2. Hamar, M., *et al.* Normothermic Ex Vivo Kidney Perfusion Reduces Warm Ischemic Injury of Porcine Kidney Grafts Retrieved After Circulatory Death. *Transplantation* **102**, 1262-1270 (2018).
3. Kaths, J.M., *et al.* Eight-Hour Continuous Normothermic Ex Vivo Kidney Perfusion Is a Safe Preservation Technique for Kidney Transplantation: A New Opportunity for the Storage, Assessment, and Repair of Kidney Grafts. *Transplantation* **100**, 1862-1870 (2016).
4. Kaths, J.M., *et al.* Continuous Normothermic Ex Vivo Kidney Perfusion Improves Graft Function in Donation After Circulatory Death Pig Kidney Transplantation. *Transplantation* **101**, 754-763 (2017).
5. Lachmann, A., *et al.* Massive mining of publicly available RNA-seq data from human and mouse. *Nat Commun* **9**, 1366 (2018).
6. Brown, K.R., *et al.* NAViGaTOR: Network Analysis, Visualization and Graphing Toronto. *Bioinformatics* **25**, 3327-3329 (2009).
7. Damman, J., *et al.* Hypoxia and Complement-and-Coagulation Pathways in the Deceased Organ Donor as the Major Target for Intervention to Improve Renal Allograft Outcome. *Transplantation* **99**, 1293-1300 (2015).
8. Tran, M., *et al.* PGC-1alpha promotes recovery after acute kidney injury during systemic inflammation in mice. *J Clin Invest* **121**, 4003-4014 (2011).
9. Ritchie, M.E., *et al.* limma powers differential expression analyses for RNA-sequencing and microarray studies. *Nucleic Acids Res* **43**, e47 (2015).
10. Kang, H.M., *et al.* Defective fatty acid oxidation in renal tubular epithelial cells has a key role in kidney fibrosis development. *Nat Med* **21**, 37-46 (2015).
11. Liu, J., *et al.* Molecular characterization of the transition from acute to chronic kidney injury following ischemia/reperfusion. *JCI Insight* **2**(2017).
12. Huang, H., *et al.* Proteo-metabolomics reveals compensation between ischemic and non-injured contralateral kidneys after reperfusion. *Sci Rep* **8**, 8539 (2018).
13. Port, J.D., *et al.* Temporal expression of miRNAs and mRNAs in a mouse model of myocardial infarction. *Physiol Genomics* **43**, 1087-1095 (2011).
14. Schneider, C.A., Rasband, W.S. & Eliceiri, K.W. NIH Image to ImageJ: 25 years of image analysis. *Nat Methods* **9**, 671-675 (2012).
15. Schindelin, J., *et al.* Fiji: an open-source platform for biological-image analysis. *Nat Methods* **9**, 676-682 (2012).
